# ChatGPT as a Tool for Biostatisticians: A Tutorial on Applications, Opportunities, and Limitations

**DOI:** 10.1002/sim.70263

**Published:** 2025-10-23

**Authors:** Dennis Dobler, Harald Binder, Anne‐Laure Boulesteix, Jan‐Bernd Igelmann, David Köhler, Ulrich Mansmann, Markus Pauly, André Scherag, Matthias Schmid, Amani Al Tawil, Susanne Weber

**Affiliations:** ^1^ Department of Mathematics RWTH Aachen University Aachen Germany; ^2^ Institute of Medical Biometry and Statistics Faculty of Medicine and Medical Center ‐ University of Freiburg Freiburg im Breisgau Germany; ^3^ Institute for Medical Information Processing, Biometry, and Epidemiology LMU Munich Munich Germany; ^4^ Munich Center for Machine Learning Munich Germany; ^5^ Department of Statistics TU Dortmund University Dortmund Germany; ^6^ Institute for Medical Biometry, Informatics and Epidemiology University Hospital Bonn Bonn Germany; ^7^ Research Center Trustworthy Data Science and Security University Alliance Ruhr Dortmund Germany; ^8^ Institute of Medical Statistics, Computer and Data Sciences Jena University Hospital ‐ Friedrich Schiller University Jena Germany

**Keywords:** causal analysis, diagnostic accuracy, generative AI, individual‐level surrogacy, large language model, latent class analysis, meta‐analysis, sample sizes planning, simulation study, translation programming languages

## Abstract

Modern large language models (LLMs) have reshaped the workflows of people across countless fields—and biostatistics is no exception. These models offer novel support in drafting study plans, generating software code, or writing reports. However, reliance on LLMs carries the risk of inaccuracies due to potential hallucinations that may produce fabricated “facts”, leading to erroneous statistical statements and conclusions. Such errors could compromise the high precision and transparency fundamental to our field. This tutorial aims to illustrate the impact of LLM‐based applications on various contemporary biostatistical tasks. We will explore both the risks and opportunities presented by this new era of artificial intelligence. Our ultimate conclusion emphasizes that advanced applications should only be used in combination with sufficient background knowledge. Over time, consistently verifying LLM outputs may lead to an appropriately calibrated trust in these tools among users.

## Abstract 2

This article is the main paper for a commentary article: https://doi.org/10.1002/sim.70271

**Abbreviations**
AI: artificial intelligence
API: application programmer interface
LLM: large language model

## 1 Introduction

In recent years, generative artificial intelligence (AI) has created numerous opportunities to enhance everyday tasks across various professions, including those of biostatistics. Large language models (LLMs) are readily available through commercial chatbots such as ChatGPT by OpenAI, Copilot by Microsoft, Gemini by Google, LlaMA by Meta AI, and DeepSeek by DeepSeek. These models generate text outputs based on input prompts using complex pre‐trained probabilistic models. As of June 12, 2025, the website huggingface.co/models lists and offers more than 240 000 pre‐trained models under the category “Text Generation”, alongside many other sub‐categories within “Natural Language Processing”. The domain of LLMs is rapidly evolving; in addition to increasing complexity, many LLMs now integrate image‐focused AI capabilities or possess the ability to write and compile software code. For example, ChatGPT has incorporated the text‐to‐image generating system DALL‐E and the text‐to‐video generator Sora, and it utilizes the programming language Python internally [1, 2].

The relevance of AI—and LLMs specifically—in medical and biostatistical fields is widely recognized beyond these professional fields. For example, magazines like Ecologist^1^ have featured articles highlighting their current applications and potential future developments.

Extensive literature exists on the use of LLMs in biostatistics. Two examples concern analyses of observational data from the National Health and Nutrition Examination Survey (NHANES) [3] and of clinical trial data [4]. Systematic studies have assessed the quality of data analyses provided by LLMs for tasks focused on coding [5], selecting appropriate tests [6] or conducting simple statistical analysis [7]. Thapa and Adhikari [8] and Clusmann et al. [9] discussed multiple opportunities, pitfalls, and potential future directions of LLMs in (bio)medical research. Within radiology research's biostatistical tasks, Ghosh et al. [10] concluded that LLMs are most beneficial for researchers with foundational knowledge in biostatistics or machine learning. Goh et al. [11] conducted a randomized clinical trial involving 50 physicians to investigate LLMs' influence on diagnostic reasoning, but they did not find significant improvements due to their use. Fichtner et al.'s study [12] examined integrating LLMs into academic statistical consulting services as supportive tools over six months to assess impacts on workflows and potential benefits. (The analysis of the study data is currently ongoing.)

Sohail [13] conducted a Scopus search revealing rapid growth in scientific publications about ChatGPT—approximately 26% related to medical science—yet an in‐depth tutorial tailored for biostatisticians remains absent. Another survey [14] identified recent trends and applications of LLMs in healthcare through analysis of 175 relevant publications.

This tutorial aims to demonstrate text‐based generative AI's potential usefulness for professional biostatisticians while highlighting caveats requiring attention. It serves as a guide (i) why LLM outputs should not be blindly trusted, (ii) in which cases they seem reliable, and (iii) potential strategies for effectively utilizing these tools to simplify work processes. We exemplify this through several use cases: Meta‐analysis, latent class analysis, individual surrogacy estimation, sample size planning, causal analyses, simulation studies, and programming language translation. Given that ChatGPT is among the most recognized generative AI tools and because of its built‐in data analysis mode, our focus is on OpenAI's GPT‐4o model with the Plus subscription, while also including targeted analyses of the newer o4‐mini variant for specific cases. This tutorial primarily targets practical biostatisticians who possess sufficient background knowledge in statistics. One of our findings is that the user must be able to verify and, if necessary, correct the output of the LLM. We view LLMs as valuable tools for streamlining many routine tasks of biostatisticians. However, this tutorial does not address regulatory aspects [15] nor does it assess their general summarization abilities, writing skills, mathematical logic, or capacity for rigorous mathematical proofs.

This paper originated from members of the International Biometric Society (German Region) interested in exploring the “Use of LLMs by Biostatisticians” within the society's initiative “AI and Biometry”. The initiative also includes focal points on “Biometry's Contributions to AI Projects” and “Teaching AI in Biometry”. These contributions complement the earlier position paper “Is there a role for statistics in artificial intelligence?” [16] authored by members of the German Consortium in Statistics (DAGStat).

This tutorial paper is organized as follows. Section 2 contains brief general descriptions of LLM functionalities and characteristics, with specific reference to OpenAI's ChatGPT. Section 3 contains exemplary use cases along with lessons learned. Finally, Section 4 re‐evaluates and summarizes our findings while discussing additional relevant aspects for biostatisticians utilizing LLMs. The Supporting Information contains all the chats we used during the preparation of the present paper. In addition, an online repository^2^ contains all relevant software code and data sets generated by GPT‐4o and, where applicable, the newer o4‐mini model.

## 2 A Few Words on LLMs in General and ChatGPT in Particular

### 2.1 LLMs in General

LLMs are text‐based machine learning models with a large number of parameters. These parameters are learned by self‐supervised training on extensive text corpora, often sourced from the internet. A notable example is *generative pre‐trained transformers* (GPTs), which are deep learning models employing artificial neural networks with multiple layers and a multi‐head attention mechanism [17].

One well‐known concern with LLMs is their handling of factual information. As complex probabilistic models, LLMs may produce *hallucinations* [18]. The model parameter known as *temperature* influences the randomness of an LLM's output; higher temperatures result in more varied outputs. Consequently, submitting identical prompts multiple times generally yields different results.

### 2.2 ChatGPT in Particular

OpenAI does not publicly disclose the training data corpora or the number of model parameters for its LLMs. Users can enhance ChatGPT's output by assigning roles and providing background information, such as acting as a biostatistician working on a Phase II trial for advanced pancreatic cancer at a pharmaceutical company. It is important to note that OpenAI may update specific versions of ChatGPT without notifying users.

A ChatGPT Plus subscription offers unlimited access to the web‐based service, while the ChatGPT Application Programming Interface (API) requires payment based on token usage. A token represents part of a sentence, such as a word or text symbol; approximately four characters (or three‐quarters of a word) constitute one token, with 100 tokens equating to roughly 75 words.^3^

For this paper's preparation, we obtained ChatGPT Plus subscriptions, which provided enhanced access to the *data analysis mode*. It is particularly beneficial for biostatistical analyses, simulations, coding tasks, and more, as it integrates Python for high‐level software execution.^4^ Additionally, it facilitates data visualizations and enables software code preparation in other programming languages like R.

By the time of writing this manuscript, the model GPT‐4o was chosen for investigating the use cases due to its unique access to the data analysis mode. However, on April 16, 2025, OpenAI introduced the newer models o3 and o4‐mini. To also evaluate their performance on certain tasks like coding or statistical analyses, we tested these models on the meta‐analysis, sample size calculation, and simulation study use cases. In this connection, we also tried the new functionalities of *deep research* (web‐retrieval) and *reasoning* in Sections 3.2 and 3.7. While *deep research* can be activated via a corresponding button, the prompted request to search through documents on the internet via web‐retrieval has the same effect.

### 2.3 How to Prompt

Prompting strategies can vary, and we will focus on two primary approaches. The first involves crafting a single, detailed prompt that outlines the general context, prerequisites, specific tasks, desired output format, and other relevant details. Alternatively, users may opt for multiple shorter prompts that iteratively provide specific details. The advantage of the latter approach is the ability to address and correct any flawed outputs on the fly. In preparing this paper, we experimented with both methods and found that they ultimately produced similar results; therefore, we do not differentiate between these prompting styles in subsequent discussions; see the screenshots of the chats related to Sections 3.3 and 3.4 below, where different prompting strategies were applied. Supporting Information includes all chat interactions used during the paper's preparation, while our online repository contains all software code and datasets generated.

In this tutorial paper, we aim to represent an average reader of *Statistics in Medicine* who works as a biostatistician. While such readers may not be familiar with every technical term, we anticipate that they possess broad background knowledge and the capability to acquire additional expertise while critically evaluating outputs from GPT‐4o.

## 3 Use Cases

We conducted a series of use cases to illustrate the typical application of generative LLMs by biostatisticians and highlight potential caveats. Specifically, we focused on tasks categorized into *reasoning and modeling* (such as 3.2 meta analyses; 3.3 latent class analysis; 3.4 individual surrogacy) or *coding and analysis* (including 3.5 samples sizes planning for survival outcomes; 3.6 causal inference; 3.7 simulation studies; 3.8 translation between multiple statistical programming languages). Each use case involved a case study using OpenAI's ChatGPT‐4o, for meta analyses, sample size planning, and the simulation study, we additionally evaluated the newer models o3 and o4‐mini. Despite the advantages of the API—such as easier control over system settings, scalability, and increased efficiency—we decided not to use it. Instead, we employed the more accessible chat‐based user interface, anticipating that most biostatisticians would prefer this option as the default.

Table 1 provides an overview of the tasks based on their (subjective) difficulty and the precision with which users specified them. It also includes the users' (subjective) overall satisfaction with the solutions provided by the LLM. Here, “users” refers to us, the team of authors; the use cases were distributed among the authors.

**TABLE 1:** Overview of use cases with categorization according to task difficulty and specification, including subjective users' satisfaction score (1: Low; 10: High).

Specification	Difficulty					
	Basic	Score	Medium	Score	Advanced	Score
Loose	—	—	Section 3.2 meta analyses (RM)	3.5	—	—
Medium	Section 3.2 data extraction in systematic reviews (RM)	7.5	Section 3.7 simulation study (CA)	7.0	Section 3.3 latent class analysis (RM)	9.0
					Section 3.4 individual surrogacy (RM)	9.0
Precise	Section 3.5 sample size planning (CA)	8.0	Section 3.8 translation between programming languages (CA)	5.5	Section 3.6 causal inference (CA)	5.0

*Abbreviations*: RM: Reasoning and modeling; CA: Coding and analysis. The scores refer to the best‐performing models used below.

**FIGURE 1:**
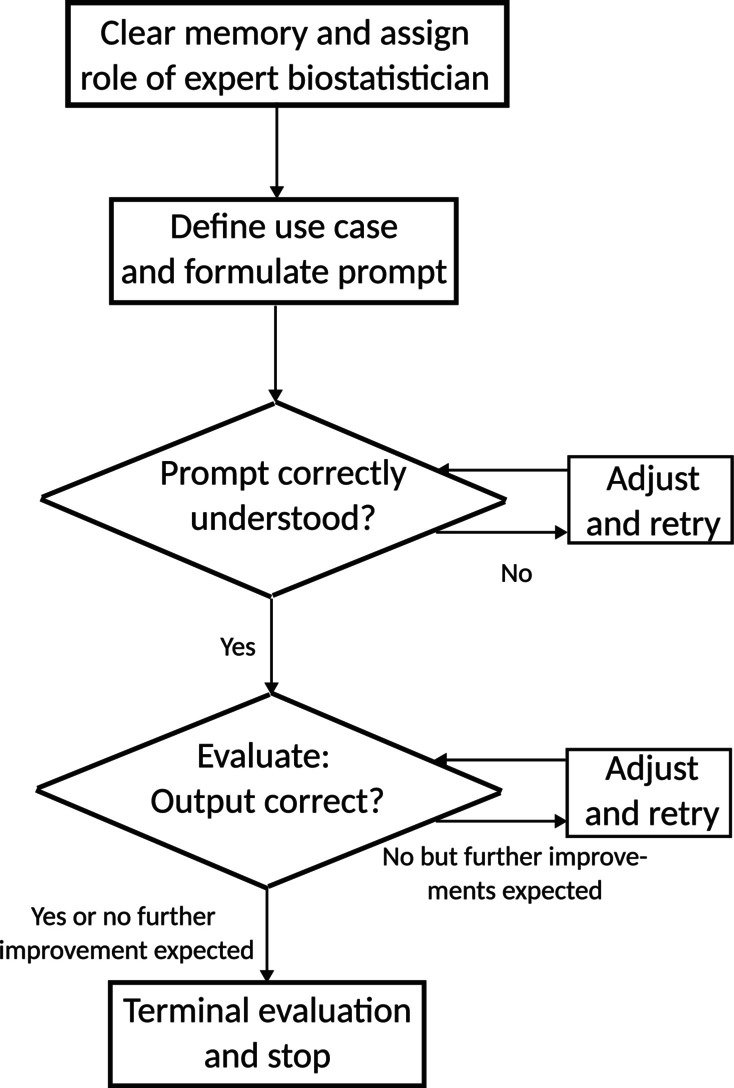
Flowchart of the use case execution, independently repeated ten times per use case.

To assess consistency in outcomes, each analysis was performed as identically as possible across ten separate chats. In each chat session or general background information setup, the LLM was instructed to assume an expert biostatistician role after clearing its memory prior to task specification. Initial prompts are detailed in corresponding sections below; however, for ease of presentation, we do not include prompts related to memory clearing or role assignment here. To maintain uniformity across sessions, additional methodological prompts were generally not allowed beyond initial instructions; minor prompts necessary for result extraction were exempted from this restriction. Figure 1 illustrates our general approach via a flowchart; minor deviations specific to certain use cases may occur.

Each use case is briefly introduced before conducting ChatGPT‐based analyses, followed by specific summaries at each subsection's conclusion; therein, we also provide various human‐in‐the‐loop checkpoints to strengthen the practical guidance—this was suggested by a reviewer.

### 3.1 Reasoning and Modeling Tasks

#### 3.1.1 Systematic Reviews and Meta‐Analyses

Systematic reviews synthesize existing research results to provide a comprehensive overview of a topic. Meta‐analysis is a specific approach that enjoys great popularity; it uses statistical methods to combine study results, thereby increasing statistical power. Methods range from simple weighted averages to more sophisticated models like random‐effects, regression, and network meta‐analyses models [19, 20, 21]. LLMs hold promise for offering valuable support in conducting systematic reviews and ensuring accurate methodological execution of meta‐analyses. These aspects are exemplarily explored in the following, using a case study on the random effects meta‐analysis of long‐term outcomes of the disease ductal carcinoma in situ—a non‐invasive form of breast cancer—conducted by Stuart et al. [22] In this use case, we evaluated both GPT‐4o and the newer o4‐mini model, each under two configurations: With reasoning mode and web‐retrieval and without both, to compare different strategies.

#### 3.1.2 Systematic Reviews

The most labor‐intensive aspect of meta‐analysis is often the systematic literature review. Guidelines for conducting meta‐analyses, such as PRISMA [23], require precise data inclusion criteria as the foundation for the literature search, making AI and LLMs promising tools for automation. While AI‐driven searches show potential [24, 25], practice shows that fully automated reviews remain challenging due to complexities in (medical) questions, the number of different literature databases, and their accessibility and LLM token limits. A promising approach is the AI‐assisted search string generation, enhancing literature search queries (e.g., keywords, MeSH terms) for databases such as PubMed [26].

A subsequent task involves extracting data from selected articles, leading to the question of whether the correct endpoints and effect sizes can be extracted by LLMs. Clear descriptions of targeted effect sizes and strict inclusion criteria are essential for this task; success also depends on how effect sizes are presented in the source paper. Using GPT‐4o, we assessed AI‐based data extraction for the studies of Vidali et al. [27] and Rudloff et al. [28] which were included in Stuart et al.'s meta‐analysis [22]. Based on this meta‐analysis, the objective was to extract the 10‐year ipsilateral local recurrence rates in cases of ductal carcinoma in situ along with basic study characteristics. We submitted the respective paper as a PDF file and a prompt with an extraction instruction, repeating the procedure ten times for each of two prompting strategies (first, with reasoning mode and web‐retrieval and, second, without) and for both GPT models (4o and o4‐mini). The resulting chats and the prompts used are included in the Supporting Information. Basic study data—such as patient age (median, min, max) and follow‐up time—were correctly extracted for most runs. However, the reported recurrence rates varied between chats, models, and prompting strategies; overall, GPT‐4o performed consistently well across the two studies and prompting strategies. With reasoning and web‐retrieval enabled, GPT‐4o identified the correct recurrence rates in all ten runs for the Vidali et al. study [27] (9.6%) and in eight out of ten runs for the Rudloff et al. study [28] (11.9%). Only for two chats, an incorrect rate of 22% was reported for the latter study. The results without reasoning and web‐retrieval were nearly identical: All ten runs were correct for Vidali et al., and nine were correct for Rudloff et al., with the one incorrect run again reporting 22%.

The results of the newer o4‐mini model showed more variation. With reasoning and web‐retrieval, the results were similar to those by GPT‐4o: The correct rate was extracted in nine out of ten runs for Vidali et al., with one run producing an incorrect rate of 8.3%. For Rudloff et al., eight runs were correct, while two reported the incorrect 22% rate. This drastically changed for o4‐mini without reasoning and web‐retrieval: The Vidali et al. rate was still correctly identified in nine runs, with one run extracting a 10% rate—likely influenced by a figure referencing a follow‐up period of 11.3 years as also used in the meta‐analysis by Stuart et al. [22] However, for Rudloff et al. only two out of ten runs yielded the correct rate; four reported 22%, three failed to return a recurrence rate at all, and one run returned results from a completely different study by Correa et al. [29].

#### 3.1.3 Meta‐Analysis

Unlike literature searches, meta‐analyses require sound statistical expertise. GPT can function as a consultant here, assisting with key methodological decisions such as choosing between fixed effects (FE) and random effects (RE) models, assessing heterogeneity and selecting suitable estimators, constructing confidence intervals (CIs) for overall effects, deciding on the application of transformations, and evaluating the need for meta‐regression or subgroup analyses.

**FIGURE 2:**
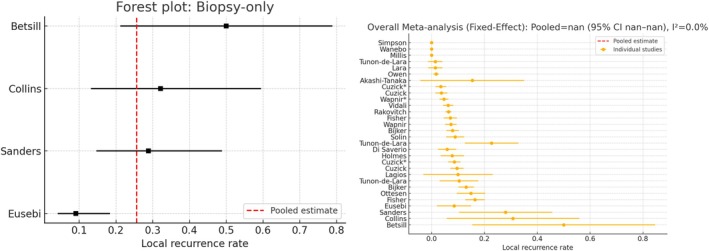
Forest plots of Chat 1 (well analyzed, treatment Biopsy‐only) and Chat 5 (erroneously analyzed, overall analysis) in the reanalysis of the meta‐analysis of Stuart et al. [22].

We tasked ChatGPT with performing a meta‐analysis based on the data from Stuart et al. [22] for invasive ipsilateral local recurrence rates across ten iterations; the full dataset (see Figure 2 therein) was provided to ChatGPT. An initial series of runs was performed with a shorter prompt and GPT‐4o. The initial prompt and the results are shown in the Supporting Information. Another ten chats were conducted with the newer o4‐mini version and a prompt asking GPT to provide more detailed reasoning, and the use of web retrieval to support methodological decisions. The results of these chats are presented below. The prompt used was as follows.

**Prompt**: *The goal is to perform a subset meta‐analysis for the given data set. The target variable is the local recurrence rate. Create a subset for all treatment types. Decide all methodological questions yourself (fixed effect vs. random effects, etc.). Follow clearly formulated rules for selecting the model type. Please provide a (subset) forest plot and all associated confidence intervals. Do not forget the pooled overall estimate and its confidence interval. For this task, you have access to up‐to‐the‐minute web retrieval to find adequate approaches for data extraction. When answering the task above, please proceed as follows*:

1. *Fetch the most relevant sources*.
2. *Show your step‐by‐step reasoning*.
3. *At the end, give me a concise answer citing the sources you used*.

The dataset's complexity arises from rates bounded between 0 and 100, with some values being zero. Appropriate preliminary steps, like zero corrections and transformations (e.g., logit, arcsine, or double arcsine), are advisable to ensure valid analysis [20, 30].

In nearly all ten independent replications, GPT‐o4‐mini's analyses appeared to be technically sound and promising. However, each of the ten runs produced distinct meta‐analysis results, and no run fully aligned with our preferred analysis approach. The pooled estimates for each treatment and applied transformations alongside model selections for all chats are detailed in Table 2. It should be emphasized that there is no single correct model. Our chosen benchmark model (see the last row of the table) is a frequentist hierarchical model that is structurally comparable to the approaches used by ChatGPT. Given the heterogeneity of the data, this model includes random effects and employs a logit transformation to appropriately model the rates.

**TABLE 2:** Obtained effect estimates for each treatment and each of the ten GPT‐o4‐mini chats, together with information about the usage of a transformation and the decision between RE and FE. In addition, the last row shows a re‐analysis using the logit transformation and hierarchical RE model structure.

Pooled est.	Mastectomy	Surg. & radiation	Surg. no radiation	Biopsy‐ only	Overall	Rate transf.	RE/FE	Decision criterion
Chat 1	0.03	0.07	0.11	0.26	0.09	Logit	Mixed	$I^{2}\geq 0.5$
Chat 2	0.02	0.06	0.11	0.26	0.08	Continuity correction for all counts	Mixed	$I^{2}\geq 0.5$
Chat 3	0.00	0.06	0.11	0.25	0.00	—	RE	$I^{2}\geq 0.5$
Chat 4	0.02	0.06	0.11	0.26	0.07	Continuity correction for zero counts	Mixed	$I^{2}\geq 0.5$
Chat 5	0.02	0.06	0.11	0.25	0.15	—	Mixed	$I^{2}>0.5$
Chat 6	0.00	0.06	0.11	0.25	0.07	Continuity correction for zero counts	Mixed	$I^{2}>0.5$
Chat 7	0.01	0.06	0.11	0.25	0.07	Continuity correction for zero counts	Mixed	$I^{2}>0.5$
Chat 8	0.00	0.06	0.11	0.25	0.00	—	RE	$I^{2}\geq 0.5$
Chat 9	0.03	0.06	0.21	0.16	0.11	—	Mixed	$I^{2}>0.5$
Chat 10	0.02	0.06	0.11	0.26	0.07	Continuity correction for zero counts	Mixed	$I^{2}\geq 0.5$
Our analysis	0.03	0.07	0.11	0.25	0.08	Logit	RE	—

Key findings of the ten runs are as follows: In 9 out of 10 runs, GPT‐o4‐mini correctly identified the hierarchical structure of the dataset, and the resulting estimates were close to our analysis. Only in Chat 9 were larger deviations at hand for four of the five treatment‐specific estimates. This could be due to confusion between the surgery without radiation and biopsy‐only subgroups. A logit transformation was applied only once. The transformation simplifies the handling of 0 event studies and prevents negative estimates or CIs. In some of the chats, where no such transformation was used, such implausible negative results appeared. A continuity correction for zero counts was applied in four chats; in one chat, a similar adjustment was made to all counts, not just zero counts. In two of the cases where no transformation was present, some of the estimates became zero due to estimation problems (Chats 3 and 8).

GPT‐o4‐mini provided both FE and RE results together with a recommendation for model selection (FE vs. RE) based on the heterogeneity statistic $I^{2}$—the percentage of variability attributed to heterogeneity. Unlike GPT‐4o (see Supporting Information), the newer o4‐mini version applied this criterion consistently across all ten chats; only the $I^{2}$ cut‐off value for applying an RE model was at least 0.5 six times and greater than 0.5 and four times. While the model clearly stated these decision criteria when prompted, its reliance on $I^{2}$ as a threshold‐based decision rule highlights a common pitfall. This criterion is usually not recommended in the literature. Instead, a decision between an FE and an RE model should be conceptually driven by the study context and underlying assumptions about effect variability [20, 31, 32]. Thus, selecting the appropriate model still requires user expertise.

Despite this variability, some runs produced fairly accurate results. An example of a rather correct first‐try analysis is given in Chat 1. The corresponding forest plot for the treatment biopsy‐only subgroup is shown in Figure 2 (left). Here, the LLM performed a logit transformation along with nearly‐correct subgroup analyses that would have led to the same conclusions. In contrast, Figure 2 (right, from Chat 5) illustrates a very problematic result where no rate transformation was applied and the computed CIs included implausible negative values. Thus, the user may be fortunate and receive a fairly accurate result (as in Chat 1), or encounter an incorrect one (as in Chat 9). The forest plots resulting from the other chats are included in the Supporting Information.

It should also be noted that the forest plots generated by GPT‐o4‐mini and GPT‐4o differ from standard presentations in the biostatistical literature, likely due to the underlying Python code used to produce them. However, both GPT versions can output high‐quality R code that can be executed locally by the user, making it possible to produce more standard visualizations. Here, popular meta‐analysis packages such as metafor [33] are well implemented.

It is important to emphasize that GPT tends to address methodological problems only when explicitly asked about. For example, for meta‐analyses with few studies, (modified) Knapp‐Hartung CIs are usually recommended due to a more reliable coverage [31, 34, 35]. In our evaluations, the GPT models did not mention these options unless specifically asked. Similarly, the application of transformations that are often recommended for rates [20, 30], such as logit or (double) arcsine, were rarely discussed without user intervention.

#### 3.1.4 Summary of the Use Case Assessment

Our analyses underline the need for expert oversight. While GPT can assist with implementation and, to some extent, also with data extraction, it does not yet reliably and critically recognize or address all methodological considerations so as to conduct a sound meta‐analysis. The observed variability—across runs, models, and prompting strategies—further underscores the importance of careful review and expert guidance when using LLMs for such sophisticated statistical tasks.

*Human‐in‐the‐loop checkpoints*

- *Data Extraction*: While LLMs can assist in identifying plausible values, all extracted data should be checked manually for correctness. When using GPT‐o4‐mini, we strongly recommend enabling reasoning and web‐retrieval.
- *Statistical Methods*: Explicitly inquire the methodological aspects relevant to the meta‐analysis. Key topics include, but are not limited to: The choice between random‐effects and fixed‐effect models, the use of data transformations, estimation procedures (e.g., between‐study variance estimation), and the construction of confidence intervals/inference.
- *Visualization*: Study visualizations of the results; even basic forest plots can reveal the LLM's failure to correctly interpret some structure in the data.

### 3.2 Latent Class Analysis – Unknown Gold Standard in Diagnostic Quality Assessment

In this use case, we evaluate ChatGPT's capability to assist a biostatistician in structuring and developing an analysis project aimed at estimating quality measures for two diagnostic tests. The objective is to obtain guidance on the theoretical background, concepts, and assumptions. Additionally, the interaction with ChatGPT should yield a log‐likelihood as a foundation for inference: Estimates and confidence intervals. Given that all parameters naturally fall within the interval [0,1], reparameterization is of interest to circumvent numerical issues associated with boundary constraints. Finally, ChatGPT is tasked with generating an R script that incorporates the analysis and can be executed in the biostatistician's working environment.

#### 3.2.1 Formalizing the Problem

The task involves estimating the sensitivities and specificities of two diagnostic tests in the absence of a gold standard. Diagnostic outcomes $\left(T_{1},T_{2}\right)$ are available within two populations with differing but unknown disease (D) prevalences—a classical problem addressed by Hui and Walter [36]. The respective log‐likelihood can be straightforwardly derived using the true yet unknown disease state as a latent variable. The key concept enabling likelihood identifiability is *conditional independence* of both tests given the true disease state: $P(T_{1},T_{2}|D)=P(T_{1}|D)\cdot P(T_{2}|D)$.

It is important to note that conditional independence may not hold; relaxing this assumption would necessitate modeling $P(T_{2}|T_{1},D)$ via logistic regression possibly incorporating an interaction between $T_{1}$ and D—introducing two additional parameters, that is, log odds ratios (log ORs). Under these conditions, likelihood maximization will have infinitely many solutions. One way to handle this problem is to calculate the maximum likelihood (ML) estimates for a given pair of log ORs within the model framework.

The use case implements the model under conditional independence while leaving exploration under a more complex modeling for $P(T_{2}|T_{1},D)$ to interested users. Assuming conditional independence, numeric methods are needed to determine the ML estimates of six parameters. Confidence intervals can be derived by calculation of the Hessian or by bootstrap strategies. The six parameters for sensitivities, specificities, and prevalences range within the interval [0,1], which can result in numerical problems; logistic reparameterization mitigates those challenges.

#### 3.2.2 Experiences Made by Using ChatGPT

The following prompts were used either as single (prompt after prompt), blocked (using thematic grouping), or total input:

**Prompt**: ‘*I would like to determine the sensitivity and specificity of two diagnostic tests. I do have the diagnostic test outcomes, but I do not know the gold standard. What can I do*?

*Please, tell me more about the latent class analysis*.

*I have two populations with two different disease prevalences. I have two diagnostic tests that are conditionally independent. I would like to get the log‐likelihood of this setting*.

*What is the Hui‐Walter paradigm?*

*I would like to see a worked example and an R script that implements this model*.

*I would like to add confidence intervals to the estimated sensitivities, specificities, and prevalences*.

*I would like to run a bootstrap version for robust confidence intervals*.

*I would like to reparametrize my model using a logistic parametrization for the prevalences, sensitivities, and specificities*.

*Please update the bootstrap function too*.

*Could you also perform the calculation of the confidence intervals via the Hessian? Please provide a comprehensive R code for the issues discussed in this session.’*

In each session, ChatGPT explains the classification of the problem in the area of latent class analysis and emphasizes the conditional independence of diagnostic tests as an important prerequisite for the analysis, referencing the article by Hui and Walter [36]. The methodological guidance provided is convincing, offering useful alternatives to ML approaches and advising on Bayesian strategies. It consistently delivers a correct log‐likelihood (LL), explains LL maximization methods, and proposes a numerical procedure for computing the negative inverse Fisher information matrix.

ChatGPT provides code for calculating 95% confidence intervals, illustrating this with examples, and also suggests using a parametric bootstrap for the interval estimation. The results of Hui and Walter can be reproduced. However, the main issue in this use case is numerical problems when executing some of the R scripts provided by ChatGPT; interestingly, these problems were not apparent during the sessions. It appears that the underlying Python procedures were more robust in addressing specific numerical challenges than corresponding R procedures; also see the use case on translation between programming languages in Section 3.8 below.

To circumvent boundary problems with the R function optim(), it is necessary to specify the lower and upper parameters; ChatGPT used 0 as lower and 1 as upper bounds, resulting in numerically unacceptable solutions. Adjusting the bounds to 0.001 (lower) and 0.999 (upper) resolved these problems. In several instances, the user needed to modify the provided R code for successful execution.

To avoid these numerical boundary problems, ChatGPT was also tasked with an analysis based on logistically transformed parameters—ChatGPT executed this flawlessly. Calculating the Hessian was sometimes numerically unstable, producing NaNs for the canonical parameters; however, ChatGPT's proposal for a parametric bootstrap procedure proved effective. With transformed parameters, confidence intervals could be calculated from the Hessian and the delta method, alongside standard errors on the probability scale; the parametric bootstrap also yielded comparable confidence intervals.

#### 3.2.3 Summary of the Use Case Assessment

ChatGPT articulated analytical principles clearly while inviting the user to explore alternative approaches; however, we did not follow the suggestion to look at Bayesian strategies. The examples provided by the AI looked reasonable. Execution of the R scripts made clear that specific parameter settings are needed to make the problem numerically work—for instance, beginners might overlook the crucial relevance of the parameters lower and upper in the function optim().

**Human‐in‐the‐loop checkpoints**:

- *Concepts*: Check whether conceptual frameworks are well reflected, and whether modeling assumptions and the theoretical background are correct.
- *Mathematics and formal derivations*: Ensure that the mathematical derivations are correctly presented.
- *R Codes*: Review and test the R codes.
- *Interpretability*: Ascertain that the outputs are interpretable and that they correspond to the theoretical expectation.
- *Robustness and alternative*: Use the offered Bayesian alternatives and check for consistency of results.

### 3.3 Individual‐Level Surrogacy (ILS) in the Context of Information Theory

Individual‐level surrogacy (ILS) in clinical trials refers to how well a surrogate endpoint (S) predicts the true clinical outcome (T) within individual patients. It measures how strongly variability in S explains variability in T within patients. Key questions arise regarding treatment decisions: Does a favorable surrogate value more likely indicate a favorable true clinical outcome? Does individual variation in S reflect individual variation in T? In linear models, the coefficient of determination $R^{2}$ is the measure providing the relevant information. Here, its generalization, the *mutual information* (MI), offers a powerful way to assess ILS beyond simple (linear) correlations. It captures any dependency between S and T. Unlike meta‐analytic approaches to trial surrogacy, information‐theoretic ILS is not part of core clinical trial methodology—thus, exploring its application through ChatGPT is valuable for experienced biostatisticians.

#### 3.3.1 Formalizing the Problem

Alonso and Molenberghs [37] proposed analyzing ILS via an information theory approach. Analysts first require guidance on relevant concepts, followed by understanding MI, which quantifies how much knowledge of one variable (S) informs about another variable (T)—essentially, the uncertainty reduction about T given S. Further exploration involves expressing MI within statistical models like generalized linear models (GLMs) and understanding its relation to the likelihood reduction factor (LRF). Next, practical scenarios necessitate calculating MI: What is the MI for a 2‐by‐2 table? Can it relate to ROC curves or positive predictive values? How does MI manifest within logistic regression or Cox proportional hazards models? In the subsequent subsection, we explore ChatGPT's guidance through these questions and the usefulness of a GPT‐4o‐generated R script with illustrative examples.

#### 3.3.2 Experiences Made by Using ChatGPT

The questions mentioned in the previous subsection motivate the following prompts, again used either as a single prompt (prompt after prompt), blocked (using thematic grouping), or total input in the ten independent chat sessions:

**Prompt**: *Can you explain to me the information‐theoretic approach to individual‐level surrogacy? Please recommend a paper that treats the subject from a predictive angle*.

*Can you explain to me the difference between the meta‐analytic approach and the information‐theoretic approach to individual‐level surrogacy?*

*What is mutual information?*

*Is there a relationship between mutual information and the deviance?*

*What is the Likelihood Reduction Factor and how does it relate to mutual information?*

*Can you help me calculate the mutual information of a 2‐by‐2 table? Could you provide me with an R script to do this calculation?*

*Is there a formal relationship between positive predictive value and mutual information?*

*Is it possible to calculate the mutual information for an ROC curve?*

*A ROC curve is defined by thresholds. Each threshold defines a 2‐by‐2 table. Can you give me a graph with the MI for each threshold in a ROC curve?*

*Can you give me an R script to compute MI across thresholds?*

*Please provide me with R code to estimate MI from score + class labels*.

*I would like to extend this concept to a binary logistic regression setting and the prevalence of the risk factor combinations. Can you give me an R script for a simple logistic regression to calculate its mutual information?*

*Can you give me an R script for a simple Cox regression to calculate its mutual information?*

*Please, provide me a comprehensive R script which contains all issues discussed in this session.'*

ChatGPT delivered a solid introduction to the methodological field, covering its key concepts and definitions. Each session provided the same formal definition of the MI, accompanied by more or less extensive explanations in lay language. In all sessions, the AI provided a helpful, simple, and tabulated summary to differentiate between meta‐analytic and information‐theoretic approaches. It was particularly adept at translating formal definitions into practical examples with corresponding R code.

However, ChatGPT's inability to supply accurate literature references was surprising; many cited articles could not be found in existing literature, and even when citations were correct, DOI identifiers were often inaccurate. Among the ten R scripts generated, one resulted in an error due to improper usage of breaks, which was subsequently corrected. The R scripts for calculating MI across different contexts—such as 2‐by‐2 tables, MI and ROC curves, MI of a logistic regression, and of a Cox regression—were consistent.

Observing how ChatGPT processed prompts proved insightful: It began with task structuring before presenting specific responses as answers, followed by a summary and an outlook on potential extensions. The initial task structuring offered valuable insights into whether the AI's response aligned correctly with the intended direction, facilitating the formulation of focused follow‐up inquiries.

#### 3.3.3 Summary of the Use Case Assessment

ChatGPT provided a good introduction to a challenging and complex field. The additionally provided suggestions for subsequent steps and post‐prompt processing were helpful—although these recommendations were not pursued further. The most unexpected limitation encountered was the system's inability to provide pertinent literature references.

*Human‐in‐the‐loop checkpoints*:

- *Check the references*: Check if recommended papers exist and discuss information‐theoretic individual surrogacy.
- *Concepts well explained*: Check whether meta‐analytic and information‐theoretic approaches are appropriately explained; also, whether the relationship between log‐likelihood improvement and mutual information is properly characterized.
- *Correct buss words*: Verify that *joint‐modeling, entropy reduction*, and *mutual information* are mentioned.
- *Formal correctness*: Confirm that the formula and the discussed derivations are correct.
- *R Codes*: Ensure that the R codes are correct and the output makes sense.
- *Robustness and alternative*: Compare results with alternative predictive metrics; use alternative packages (scoringRules) to get a deeper understanding.

### 3.4 Coding and Analysis Tasks

#### 3.4.1 Sample Sizes Planning for Survival Outcomes

LLMs offer various applications in the context of study planning and data analysis. Beyond calculating required sample sizes, LLMs can assist in preparing these calculations and generating structured text for study protocols regarding sample size planning.

#### 3.4.2 Aspects for Sample Size Calculation in Survival Analysis

Unlike study designs with fixed time outcomes, survival analysis focuses on time‐to‐event data, necessitating specific considerations for sample size calculation. In addition to standard parameters—significance level (α) and power (1−β)—factors such as the expected number of events, follow‐up duration, and censoring must be accounted for. The chosen statistical method—often log‐rank tests or Cox regression—also influences the required sample size. For detailed guidance on sample size calculations in proportional hazards models, refer to Schoenfeld's work [38].

#### 3.4.3 Example

In this use case, we consider a treatment comparison in a survival framework: A new drug for the treatment of advanced pancreatic carcinoma has been developed; to assess its efficacy compared to standard therapy, a study is planned with overall survival as the primary outcome. Consequently, a sample size calculation is necessary.

As biostatisticians, we are familiar with the methodological requirements for such a calculation and have already discussed relevant parameters with the clinical investigators. GPT‐4o is employed in this scenario to support the following tasks: (i) conducting the sample size calculation based on predefined assumptions; (ii) generating appropriate text for inclusion in the study protocol; and (iii) simulating corresponding data to aid statistical analysis preparation. Our use case‐specific prompt contained the sample size calculation task:

 
**Prompt**: ‘*I would like to compare a new therapy with the standard therapy regarding survival in advanced pancreatic carcinoma. Please perform a sample size calculation and incorporate the following parameters*:
  · *Primary outcome: Overall survival*.
  · *Median survival time in the standard therapy group: 12 months*.
  · *Median survival time in the new therapy group: 18 months*.
  · Power=80%(1−β=0.8)
  · *a two‐sided significance level of* α=0.05.
  · *The allocation ratio should be 1:1 (equal group sizes)*.
  · *Patients will be followed up for 2 years*.
  · *We expect a dropout rate of 15%*.
 
*Use the data analysis mode*.’

While we do not focus on the correctness of the specific assumptions provided, our emphasis is on the correctness and consistency of ChatGPT's responses across the ten sessions. Each chat followed the same prompt guideline to ensure comparability of outputs; see the Supporting Information for details. The initial prompt for the sample size calculation provides clear input about the required parameters, which is then followed by a series of interactive and logically structured prompts.

Assuming an exponential distribution, the median survival times provided correspond to hazard rates of 0.0578 for the standard treatment group and 0.0385 for the new treatment group, resulting in a hazard ratio (HR) of 0.6667. Using this information alongside group allocation ratios, the required event numbers were calculated according to Schoenfeld's method [38]. Incorporating the planned follow‐up period and the anticipated dropout rate, the probability of an event can be estimated using the exponential survival function. This enables the determination of the overall sample size needed. Using the power logrank command in STATA/SE 17.0 and Schoenfeld's method, the given parameters yield an estimated overall sample size of 334, that is, 167 per group.

Running only the first three prompts (without further interaction) ten times resulted in five different estimates of the overall sample size (min=78, 1st quartile=83, median=84, 3rd quartile=84, max=665). Chat 6 was the only chat with a sample size greater than 88. ChatGPT usually started with the calculation of the number of events needed, followed by the calculation of the overall group size, incorporating the duration of follow‐up and the dropout rate. Some of the variability in the reported sample sizes may be attributed to rounding during intermediate steps of the calculation.

**FIGURE 3:**
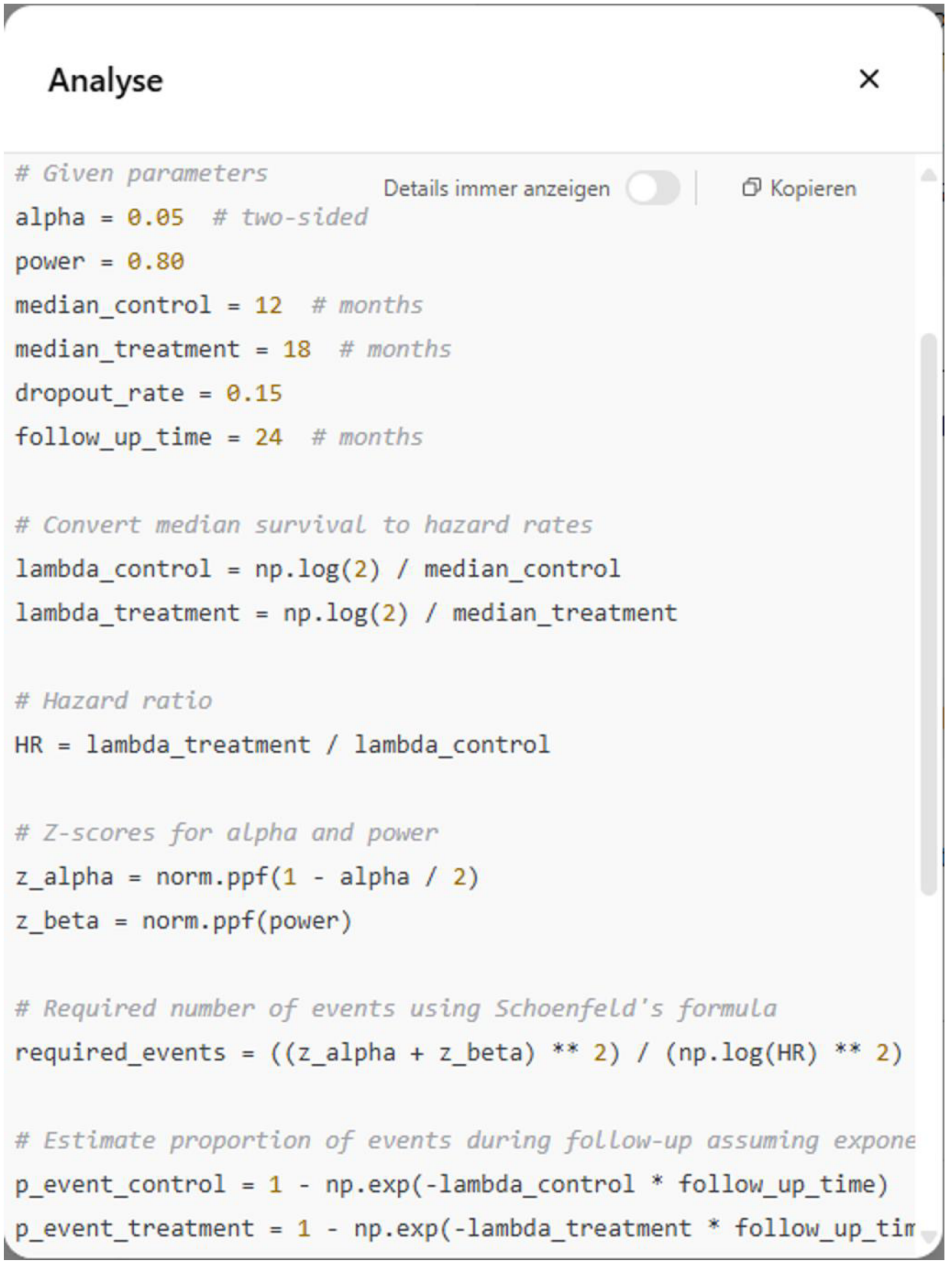
Code snippet from Chat 3 showing the calculation of the required number of events in the data analysis mode of GPT‐4o without accounting for group proportion.

Checking the complete explanations of each step in the sample size calculation performed, provided by ChatGPT upon request, and a look into the compiled Python code allowed for an evaluation of whether the sample size calculation was valid and, if not, where the calculation went wrong. In each run, ChatGPT had trouble with the determination of the number of required events. ChatGPT consistently failed to incorporate group proportions in the denominator when applying Schoenfeld's formula [38], leading to underestimated event counts. A screenshot of the corresponding Python code compiled in Chat 3 is shown in Figure 3. Note that, when ChatGPT explained the sample size steps, the results of intermediate calculations provided as text might not correspond to the actually calculated intermediate results; compare with the explanation of the initial sample size calculation in Chat 10 for the estimated event probability in 24 months; see the Supporting Information for a screenshot of the chat.

Only Chat 6 initially incorporated the group proportion correctly. However, in this chat, ChatGPT considers the overall number of events needed as the events needed per group, and hence effectively doubles the required number of events, resulting in an overestimated sample size. After prompting ChatGPT to check the calculation of the required number of events and whether group proportions were correctly accounted for, this led to a correction in each chat. The revised estimates resulted in six different values (min=311, 1st quartile=332,median=333, 3rd quartile=334, max=350), which were all close to the sample size calculated via STATA.

The paragraphs for the study protocol provided by ChatGPT were comprehensive, incorporating the assumed parameters and explanations on how the sample size was determined. Simulation of the corresponding data to prepare the data analysis worked straightforwardly. ChatGPT incorporated risk factors suitable for the setting of pancreatic carcinoma. For instance, the Eastern Cooperative Oncology Group (ECOG) performance status was simulated in each chat. The ECOG performance status is known to be a prognostic factor in pancreatic cancer [39, 40].

**FIGURE 4:**
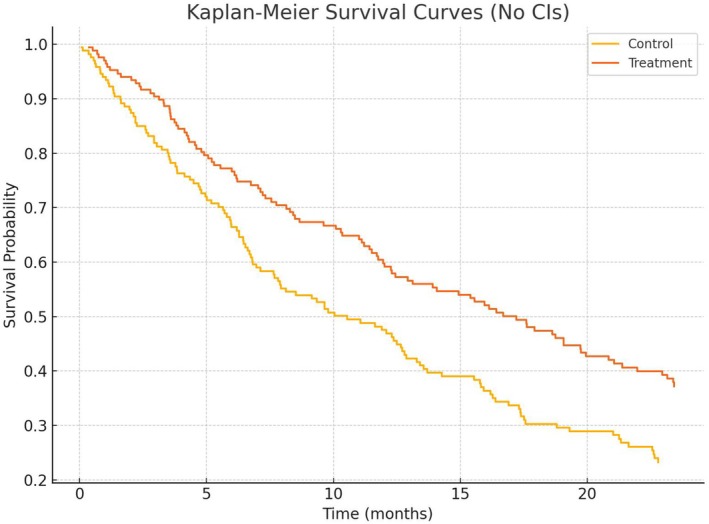
Initial Kaplan Meier curves given by ChatGPT in Chat 9. The curves do not start at time zero with 100%.

When asking for a visualization of the data, usually Kaplan–Meier (KM) curves are provided. In three of ten chats, KM curves did not start at time zero with the value 100% (Chats 7, 9, 10). In Figure 4, the initial KM plot of chat 9 is shown.

Performing the Cox regression caused some troubles in producing the results table, which were usually solved by ChatGPT without further interaction.

When asking ChatGPT to provide the code needed to run the analysis in R, the returned code worked without any corrections in seven of the ten chats. In Chats 3, 6, and 10, however, there were some issues with producing the plot or the results table. In Chat 7, the simulation of the data was included in the R code.

#### 3.4.4 Results Based on Newer ChatGPT Models

To compare ChatGPT's analytical results across different models, we additionally ran three chats per newer version (o3 and o4‐mini) following the initial prompts of the prompt guideline to evaluate whether the estimated sample sizes would more closely approximate the one obtained using Schoenfeld's formula (reference value obtained via STATA/SE 17.0 is an overall sample size of 334). Recall that the initially returned sample size estimations with the 4o version resulted in five different numbers (min=78, 1st quartile=83, median=84, 3rd quartile=84, max=665). The resulting overall sample sizes after the initial estimation via the O3 version are 84, 84, and 333, respectively. In one of the three runs, the group allocation was incorporated correctly (Chat 3). In one of the remaining two runs, it took ChatGPT o3 almost five minutes to check and correct this (Chat 1). On the other hand, ChatGPT o4‐mini initially returned the overall sample sizes 334, 334, and 81, respectively. Only the third chat did not incorporate the group allocation correctly. Overall, ChatGPT o4‐mini (<26 s each) was much faster in providing a first estimation of the sample size than ChatGPT o3 (>43s each). In summary, based on three runs with the newer model, ChatGPT o4‐mini outperformed both ChatGPT o3 and GPT‐4o by providing more accurate initial sample size estimates.

#### 3.4.5 Summary of the Use Case Assessment

While ChatGPT was able to provide complete explanations, the sample size calculations showed substantial variability across sessions, with only partial adherence to established methodology such as Schoenfeld's formula. Specifically, a systematic error in the denominator was observed in all sessions, failing to account for group allocation proportions. After pointing this out to ChatGPT, the corrected sample sizes ranged from 311 to 350 compared to 334 from STATA 17.0. Some of the observed variability may also be attributed to rounding during intermediate steps. The generation of protocol text and simulated data was successful, and Kaplan–Meier visualizations could be obtained with minor limitations. The ability to generate functional R code for analysis varied, but was generally acceptable. Overall, ChatGPT can be a helpful tool for support with such routine biostatistical tasks, but expert oversight is essential to ensure methodological correctness.

*Human‐in‐the‐loop checkpoints*:

- *Check formula considered by ChatGPT*: Ensure that the suggested formula aligns with standard methodology—for example, Schoenfeld's formula for survival data—as deviations or oversimplifications are common and may go unnoticed without domain knowledge.
- *Ensure correct incorporation of assumed parameters into the sample size calculation*: For instance, verify if group allocation ratios, effect sizes, power, and significance level are appropriately accounted for.
- *Run several chats to check consistency in the returned sample sizes*: Variability across sessions may indicate mistakes in ChatGPT's approach and can help to identify where corrections are needed.

### 3.5 Causal Inference and the Use of IPCW to Adjust for Treatment Switching

#### 3.5.1 Causal Estimands and Intercurrent Events

Randomized controlled trials estimate causal treatment effects by comparing outcomes across different treatment arms, assuming that the initial balance achieved through randomization holds throughout follow‐up [41]. This assumption may be disrupted by intercurrent events (ICEs) such as cross‐over, treatment discontinuation, initiation of second‐line therapy, or death [41, 42]. When such events occur, they may compromise the interpretability of the outcome or preclude its observation [43], posing challenges for valid causal inference [42]. Crossover is particularly common in oncology trials, where control‐arm patients are often permitted to cross‐over to the experimental treatment—typically after disease progression—once interim analyses suggest a potential benefit [44, 45]. Despite its potential to bias estimates, treatment crossover is typically not addressed in the intention‐to‐treat (ITT)‐based primary analysis of overall survival. This leads to two key limitations: First, the potential benefits received by those who crossed over to the experimental treatment are not properly accounted for, typically resulting in an underestimation of the experimental treatment effect [46]; second, ignoring the cross‐over behavior—that would not occur outside the trial setting—limits the relevance of trial findings for real‐world decision‐making [47]. It is therefore of interest for different stakeholders to explore causal effects under hypothetical strategies that prohibit treatment cross‐over [48]. Addressing this requires a principled methodological approach for defining, estimating, and interpreting treatment effects in the presence of ICEs.

#### 3.5.2 Example

In this use case, we examine a scenario commonly encountered in oncology trials, where a substantial proportion of patients with non‐small cell lung cancer switched to the experimental treatment following disease progression. Our objective was to estimate the causal effect of sustained treatment intake with the experimental versus control therapy on overall survival, under a hypothetical strategy in which crossover would not occur. Answering this question requires a combination of causal reasoning and statistical modeling, particularly in the presence of time‐varying confounding affected by prior treatment intake. Here, we explored the use of the inverse probability of censoring weighting (IPCW) approach to estimate causal treatment effects; we evaluated ChatGPT as a tool to support our analytical process, in particular, with the following tasks:

a. defining the target estimand according to the ICH E9(R1) framework [43];
b. identifying methodological limitations of standard statistical approaches and motivating the use of advanced causal methods such as IPCW;
c. Depicting causal relationships using a directed acyclic graph (DAG);
d. interpreting relevant literature;
e. implementing the IPCW approach in R.

The initial prompt used for all runs is as follows:

**Prompt**: *‘I have data from a randomized clinical trial. I want to compare the efficacy of the experimental treatment with that of the control on overall survival in patients with non‐small cell lung cancer. In this two‐arm RCT, a considerable number of patients in the control arm switched to the experimental treatment after disease progression, while those in the experimental arm never switched. My goal is to estimate the causal effect of sustained intake of the experimental treatment versus sustained intake of control on Overall survival, had the control group patients not been allowed to switch. Based on the information provided above, please define my target Estimand, following the guidance from the Estimand framework (ICH‐E9 addendum).’*

A key methodological challenge in estimating causal effects—formalized by the target estimand described in the initial prompt—is the presence of treatment‐confounder feedback, whereby time‐varying covariates, such as ECOG performance status or progression, not only influence treatment decisions over time, but are also affected by prior treatment. This structure invalidates the use of standard time‐dependent Cox models [49], necessitating alternative approaches such as g‐methods, including IPCW [50]. We then instructed ChatGPT to represent the causal structure using a DAG, and later supplied a screenshot of a more carefully specified DAG to guide subsequent responses. Based on this input, ChatGPT was asked to provide a suitable IPCW analysis approach in R. In this setting, IPCW should reweight the risk sets to construct a pseudo‐population that emulates a scenario in which no cross‐over occurs [47, 51].

Here, we do not evaluate the plausibility of the assumptions made with the IPCW approach, but rather assess the methodological consistency and correctness of ChatGPT responses across all ten sessions. To allow direct comparison across sessions, all sessions were initiated with identical prompts and followed a fixed prompt sequence. We did not intervene during the sessions, as doing so would have introduced unnecessary complexity without potentially affecting subsequent outputs. Table 3 displays for some key criteria whether they were satisfied within each chat.

**TABLE 3:** Overview of fulfilled criteria across the ten GPT‐4o chat sessions for the causal inference use case.

Chat	Target estimand defined	Causal methods motivated	DAG showing treatment‐ confounder feedback	Literature correctly interpreted	IPCW correctly implemented
Chat 1	✓	✓	×	×	×
Chat 2	✓	✓	✓	×	×
Chat 3	✓	✓	×	×	×
Chat 4	✓	✓	✓	×	×
Chat 5	✓	✓	✓	×	×
Chat 6	✓	✓	×	×	×
Chat 7	✓	✓	×	×	×
Chat 8	✓	✓	×	×	×
Chat 9	✓	✓	✓	×	×
Chat 10	✓	✓	✓	×	×

In the clinical context under consideration, only control‐arm patients who progressed were permitted to switch to the experimental treatment. Our objective was to estimate the causal effect of sustained treatment strategies under a hypothetical scenario in which cross‐over does not occur. Based on this objective, ChatGPT consistently returned a correct definition of the target estimand, structured according to the five attributes outlined in the Estimand framework [43]. This was successfully achieved in all sessions. Each session included an explanation of the time‐varying confounding structure influenced by prior treatment, illustrated with examples. In all chats, ChatGPT correctly identified this as “treatment‐confounder feedback” and acknowledged that standard approaches, such as time‐dependent Cox regression, yield biased estimates in this context. Consistently, appropriate causal methods were recommended, including inverse probability of censoring weighting (IPCW), marginal structural models (MSMs), and G‐estimation of structural nested models.

However, when prompted to generate a DAG reflecting the described treatment‐confounder feedback structure, ChatGPT failed to capture the feedback mechanism in 5 of 10 sessions. In all sessions, the graphical output lacked clarity and rigor, making it unsuitable for use in scientific writing. We also assessed ChatGPT's ability to summarize foundational literature. When asked to explain the IPCW method introduced by Robins and Finkelstein [52], all sessions provided a broadly consistent and relevant explanation of the core methodological concept. However, when prompted for study‐specific details, such as treatment arms, endpoints, and the type of intercurrent events, hallucination behavior was observed in all sessions. Despite understanding the IPCW approach conceptually, ChatGPT's implementation quality varied considerably. All chats returned R code that followed a two‐step approach: First, a pooled logistic regression model (weighting model) was used to estimate the probability of remaining unswitched; and second, an outcome model was fitted to the reweighted data. Before this prompt, we had provided a deterministic switching rule—only control‐arm patients are eligible to switch following disease progression—and provided a screenshot of a detailed DAG. In this context, we expected ChatGPT to either (i) include the treatment arm as a covariate in the weighting model, or (ii) restrict the weighting model to the control group. Only 2 sessions acknowledged the first strategy, while only 1 implemented the second; the remainder did not account for this distinction. The role of post‐progression covariates in the censoring model was explicitly considered in 2 sessions. In 5 sessions, weights were calculated based on cumulative probabilities. Stabilized weights were calculated in 4 sessions, using a baseline‐only or time‐only numerator model; one session applied an overly simplistic and potentially problematic truncation rule. For the outcome model, 2 sessions correctly restricted the data to uncensored observations, while the others either included all data (including post‐censoring data) or only considered the last weighted observation. Overall, we observed a considerable variation in the quality of IPCW implementation in R, which ranged from poor to adequate. No session produced a fully correct and ready‐to‐use analysis code.

#### 3.5.3 Summary of the Use Case Assessment

Across ten sessions, ChatGPT consistently recognized the core challenges associated with estimating causal effects in the presence of treatment switching and time‐varying confounding. While all sessions correctly identified the need for IPCW and were able to outline its conceptual implementation, there was substantial heterogeneity in the representation of causal structures, the construction of weights, and the accuracy and completeness of the R software. As with other advanced causal inference methods, expert oversight remains essential to ensure correct implementation.

*Human‐in‐the‐loop checkpoints*:

- *Causal DAG specification*: Review and correct LLM‐generated DAGs for clarity, completeness, accuracy, and inclusion of treatment‐confounder feedback. Validate the temporal ordering of variables in the DAG.
- *Literature Review*: Cross‐check any literature cited or summarized by the LLM for accuracy and relevance. Confirm that key references (e.g., by Robins and Finkelstein [52] on IPCW) are correctly interpreted and contextualized.
- *IPCW weighting model*: Check inclusion of appropriate baseline and time‐varying confounders. Verify correct handling of treatment‐arm‐post‐progression specific switching eligibility (e.g., restricting censoring model to control arm after progression). Ensure that stabilized weights are correctly computed, with a reasonable numerator model specification.
- *IPCW outcome model*: Confirm proper definition of risk sets and accurate identification of censoring times.

### 3.6 Simulation Study to Evaluate the Coverage Probability of a Confidence Interval

Another frequently performed task of biostatisticians is the coding of simulation studies. Sample size planning—in particular, those for complex designs with many parameters—is one of the most frequent applications. As sample size planning was already addressed in Section 3.5 above, we decided to run a Monte Carlo simulation study to assess the coverage probability of a confidence interval. The setting was a case‐control study in which we asked for a suitable exact confidence interval for the point estimate of a risk factor effect. We asked for ten different scenarios with varying (small) sample sizes generated under the null hypothesis.

**Prompt**: *‘The goal is to perform a simulation study to assess the coverage probability of a confidence interval with nominal coverage 95%. The setting is a case‐control study design, which is a retrospective observational study that compares individuals with a disease (cases) to those without it (controls) to identify potential risk factors or exposures associated with the disease. We aim at the coverage probability of an appropriate effect size estimate in a parametric model. Propose a suitable exact confidence interval and investigate its empirical coverage under the null hypothesis of no effect. Use 5000 Monte Carlo runs, and the sample sizes must vary between 20 and 40. Provide results for ten different settings with different sample sizes and marginal probabilities. Be concise in your answer and do not provide unnecessary descriptions.’*

Each of the sessions was evaluated based on six criteria (all yes/no). First, we assessed whether the prompt functioned correctly from the outset. Second, we documented if ChatGPT suggested an exact confidence interval (CI) right from the start and, if so, which CI was reported. Third, we checked if the implementation of the simulation was (roughly) correct and, fourth, if the reported coverage was (roughly) plausible (approximately 95%). Fifth, we reviewed whether the required ten simulation settings were met. Finally, we ascertained whether ChatGPT provided correct comments or interpretations of the results. The results of the ten independent chat sessions submitted to GPT‐4o with the identical prompt are summarized in Table 4. Based on the results, we also decided to add three additional chat sessions (Chats 11–13 in Table 4) using GPT‐o3 + *deep research*.

**TABLE 4:** Overview across the ten GPT‐4o (Chats 1–10) chat sessions performing Monte Carlo simulation studies to assess the coverage probability of an exact confidence interval under no association in a case‐control design. Chat sessions 11–13 refer to results of GPT‐o3 + *deep research*. A slash (“/”) indicates that the assessment was not possible (e.g., no code was provided, but a table was presented).

Chat	Prompt working	Exact CI chosen	Correct implementation	Plausible coverage	Setting requirements met	Correct comments
Chat 1	×	×	✓	×	✓	✓
Chat 2	✓	×	✓	✓	✓	✓
Chat 3	×	×	✓	/	✓	/
Chat 4	✓	×	/	✓	✓	✓
Chat 5	×	×	✓	✓	✓	✓
Chat 6	×	×	✓	✓	×	✓
Chat 7	✓	×	✓	✓	✓	✓
Chat 8	×	×	✓	/	✓	✓
Chat 9	×	×	✓	✓	✓	✓
Chat 10	×	×	✓	/	×	/
Chat 11	✓	✓	✓	✓	✓	✓
Chat 12	✓	✓	✓	✓	✓	✓
Chat 13	✓	✓	✓	✓	✓	✓

#### 3.6.1 Summary of the Use Case Assessment

Initially, we did not require an exact confidence interval in the prompts. This resulted in quite similar (and largely correct) results after some prompt tests, since our original attempts often yielded 0% coverage. The request for exact confidence intervals turned out to be difficult for GPT‐4o; none of the ten chats provided an exact CI. Exploring this issue beyond the ten chats (and less systematically) at least resulted in some feedback by ChatGPT; for example, that a Firth correction should be applied. In Chats 11–13, in which the same prompt was used with GPT‐o3 + *deep research*, the results were significantly better. However, the initial prompt always resulted in additional requests that required some prior knowledge in biostatistics.

Conversely, the code to implement the Monte Carlo simulation to investigate the coverage probability was correct in nearly all instances. Similarly, if coverage probabilities were provided, they were plausible. ChatGPT correctly met the simulation setting requirements. Finally, while the comments and interpretations offered by GPT‐4o were largely correct, they tended to be quite generic. For GPT‐o3 + *deep research*, the interpretation was more detailed (e.g., indicating that exact confidence intervals may sometimes be conservative) and relatively similar—at the level of detail—across all three chats.

*Human‐in‐the‐loop checkpoints*:

- *Prompt engineering*: LLMs seem to support simulation studies relatively well—at least with the latest models that include reasoning. However, if the prompt is vaguely formulated or the LLM is left to make certain selections independently, this leads to additional queries in the model. The quality of the results then depends on the user's answers to these questions.
- *Code monitoring*: GPT‐4o sometimes generated pseudo‐code, whereas GPT‐o3 + *deep research* provided commented code that was better suited for subsequent work. In both cases, however, additional code development or code monitoring is recommended.
- *Result presentation*: Requirements for the table presentation were largely met—but it is recommended to verify the (face) validity of the results.

### 3.7 Translation Between Multiple Statistical Programming Languages

In collaborative research projects, analyses are often carried out in different systems by researchers with expertise in varying statistical programming languages. Certain tasks are only implemented in a specific software, while some programming languages have superior computational speed compared to the language a researcher is most proficient with. Thus, it is often desirable to translate statistical code into other programming languages. This translation process can be a time‐consuming, tedious task that involves the search for comparable software packages for specific use cases, adaptation of language‐specific syntax, and careful consideration of different function arguments and nomenclature.

The access of LLMs to software documentation and forum discussions makes them promising to reliably automate this task. This capability is explored in this section by examining ChatGPT's ability to translate statistical models of intermediate complexity into Python and R and comparing the results.

We considered fitting a logistic regression model with data‐driven feature selection for the Breast Cancer Wisconsin dataset [53]. The dataset contains 569 instances of breast mass fine needle aspirates (FNA). From each FNA, 30 continuous features were computed and used as candidate features for logistic regression. The dataset contains 212 malignant cases and 357 benign cases. A stepwise regression model was fitted that started from an empty feature set and iteratively added or removed features to optimize a certain criterion. The specific criterion varied between applications and programming languages and is further described in the following sections.

#### 3.7.1 Translating R Into Python

We specified a model that uses AIC minimization for feature selection. This is implemented in the stepAIC function of the MASS package [54] in R, while at the time of writing, no implementation corresponding to AIC‐based feature selection existed in Python. The data were randomly split into a training and a test set (80% and 20% of cases, respectively). Model evaluation was performed on the test set. For this use case, the used prompt simply asked to translate the provided R code into Python without further explanation or specification. The prompt was replicated ten times, each time using the same training/test split. To evaluate ChatGPT's ability to truthfully transfer the specific statistical model to R, several aspects were considered, such as model accuracy, features in the final model, inclusion/exclusion criteria in the stepwise feature selection, and the code's general ability to compile without further amendments.

**Prompt**: ‘*Translate the following code into python*:

*library(MASS)*

*set.seed(1)*

*temp* ← *tempfile()*

*download.file*(“https://archive.ics.uci.edu/static/public/17/breast+cancer+wisconsin+diagnostic.zip”, *temp*) *data* ← *read.table(unz(temp, “wdbc.data”), sep = “,”)*

*data* $ *V2* ← *data* $ *V2 == “M”*

*unlink*(*temp*)

*train*_*index* ← *sample*(*seq*_*len*(*nrow*(*data*)), *round*(*nrow*(*data*) · *0.8*))

*train* ← *data[train*_*index*, −1]

*test* ← *data*[‐*train*_*index*, −1]

*base*_*model* ← *glm*(*V2* ∼ *1, data = train, family = binomial*)

*step*_*model* ← *stepAIC*(

       *base*_*model*,

       *direction = “both”*,

            *scope = list*(

            *lower = V2* ∼ *1*,

            *upper = V2* ∼ *V3 + V4 + V5 + V6 + V7 + V8 + V9 + V10 + V11 +*

             *V12 + V13 + V14 + V15 + V16 + V17 + V18 + V19 + V20 + V21 +*

            *V22 + V23 + V24 + V25 + V26 + V27 + V28 + V29 + V30 + V31*

     )

)

*summary*(*step*_*model*)

*test*_*pred* ← *predict*(*step*_*model, test, type = “response”*)

*sum*((*test*_*pred* >0.5)== *test* $ *V2*)/*nrow* (*test*)

*names*(*coef*(*step*_*model*))’

In six out of ten runs, the code provided by the LLM compiled without further need for debugging. In the four cases where the code was erroneous, all chats resolved the issue by simply naming the lines that caused errors and providing the error messages. One error was caused by the use of a deprecated naming convention. Further errors were caused by numerical issues regarding feature collinearity arising in specific train/test splits. While these cases were easily solved with further ChatGPT prompts, this demonstrates the need for special consideration of input constellations that may lead to exceptional errors, which might not be covered in the original prompt.

Notably, only two of the ten chats offered an AIC‐based feature selection. Two chats relied on the SequentialFeatureSelector (SFS) method, which by default uses accuracy as a scoring metric. In one of the SFS‐based solutions, however, ChatGPT implemented a custom scoring metric that uses AIC. The other chats relied on p values for feature selection. This discrepancy in selection criteria was, however, explicitly stated in the reply in all eight chats. Seven chats offered to provide an alternative AIC‐based implementation. The success of each run in accurately implementing the specified model is summarized in Table 5.

**TABLE 5:** Results of translating a logistic model with AIC‐based stepwise feature selection from R into Python. A slash (“/”) indicates that no debugging was necessary after successful compilation.

Chat	Functional code	Successful debugging	Selection metric	AIC‐based alternative	Out‐of‐sample accuracy
Chat 1	✓	/	p value	✓	0.9737
Chat 2	✓	/	p value	×	0.9737
Chat 3	×	✓	p value	✓	0.9737
Chat 4	×	✓	p value	✓	0.9737
Chat 5	✓	/	p value	✓	0.9737
Chat 6	×	✓	AIC	/	0.8859
Chat 7	✓	/	p value	✓	0.9737
Chat 8	✓	/	p value	✓	0.9737
Chat 9	×	✓	p value	×	0.9737
Chat 10	✓	/	AIC	/	0.9737

The last column indicates a homogeneous out‐of‐sample accuracy for 9 out of 10 models. Moreover, most models selected the same features, except for chats 6, 7, and 10. However, the features chosen by these models differed from those selected by the R model. Thus, ChatGPT cannot exactly replicate results after translation into a different programming language. This is most likely due to a different handling of random number generation and/or libraries for the numerical backend of model fitting. Validation metrics, debugging messages, and output formats were consistent between the provided R implementation and all LLM‐generated Python implementations without further specification other than the original code.

Only one of the ten chats mentioned the potential need for manual package installation in Python. The generated code was documented and explained in all prompt iterations. However, despite ChatGPT correctly stating that the models do not optimize w.r.t. AIC in the chat replies, several generated code documentations wrongfully stated that AIC had been used for feature selection.

#### 3.7.2 Translating SPSS Into R

We further investigated the capability of ChatGPT to generate R code based on SPSS syntax. The graphical interface and wide range of statistical methods in SPSS make it a commonly used tool for clinical researchers. However, using a free, open‐source software may increase reproducibility, and the script‐based workflows and dynamic extension of the R language through user‐provided packages enable more collaborative work and application (and implementation) of novel methods. Again, a logistic regression model with forward stepwise selection was applied to the Wisconsin breast cancer dataset. Starting with an empty feature set, new features were added if the p value of the likelihood ratio test was lower than 0.05, whereas features with p>0.1 were removed from the model. The code was supplied to ChatGPT in SPSS syntax. In the prompt, only the task of translation into R was specified without giving further context on the syntax. The ability to run the code as‐is, selected coefficients, and truthfulness to the original code were considered for evaluation.

**Prompt**: ‘*I am running the following analysis in SPSS*:

*DATASET ACTIVATE DataSet5. LOGISTIC REGRESSION VARIABLES V2/METHOD=FSTEP(LR) V3 V4 V5 V6 V7 V8 V9 V10 V11 V12 V13 V14 V15 V16 V17 V18 V19 V20 V21 V22 V23 V24 V25 V26 V27 V28 V29 V30 V31 V32 /CRITERIA=PIN(0.05) POUT(0.10) ITERATE(20) CUT(0.5)*.

*Translate this into R code*.

*The data is loaded into R with*

*temp* ← *tempfile()*

*download.file*(“https://archive.ics.uci.edu/static/public/17/breast+cancer+wisconsin+diagnostic.zip”, *temp*)

*data* ← *read.table(unz(temp, “wdbc.data”), sep = “,”)*

*data* $ *V2* ← *data* $ *V2 == “M”*

*unlink(temp)*’

All generated scripts used either the step or stepAIC R functions, both of which are AIC‐based forward selection methods instead of likelihood ratio tests at the above p value thresholds. One query offered an additional alternative implementation using p values for feature selection in the first reply. Of the remaining 9 models, 7 explained this discrepancy in the chat and offered to provide more truthful custom implementations on request. In two of those alternatives, inclusion was not correctly implemented, so that no features were selected. Further investigation showed that this was the result of a change in the functionality of the used packages, highlighting the issue of unspecified package versions in the generated R code. A summary of code errors, the used evaluation criterion, and overlap in selected features with the SPSS model is given in Table 6.

**TABLE 6:** Results of translating a logistic model with LR‐test‐based stepwise feature selection from SPSS into R. Column “Overlap SPSS” shows the fraction of selected features in the SPSS model that were also selected in the model generated by the respective chat.

Chat	Functional code	Selection metric	LR‐based alternative	Overlap SPSS
Chat 1	✓	AIC	✓	75%
Chat 2	✓	AIC	×	62.5%
Chat 3	✓	AIC	✓	62.5%
Chat 4	✓	AIC	✓	62.5%
Chat 5	✓	AIC	✓	62.5%
Chat 6	✓	AIC	✓	0%
Chat 7	✓	AIC	×	0%
Chat 8	✓	AIC & p value	✓	62.5%
Chat 9	✓	AIC	✓	62.5%
Chat 10	✓	AIC	✓	62.5%

While the exact feature set obtained from SPSS was not recovered in any of the chats, the majority of the chats selected a consistent set of features that had moderate overlap with the SPSS model (5 out of 8 features selected in the SPSS models were selected in 80% of chats). The consistency of the results, combined with the high correlation between features, suggests that this was not due to inherent variability of the solutions provided by ChatGPT, but rather by differences in the numerical backend of model fitting.

Only one out of the ten chats covered the installation of uninstalled R packages. Although the prompt did not explain the data structure except for the name of the outcome variable, all generated scripts were compatible with the input format of the used data. All chats generated code documentation through additional comments.

#### 3.7.3 Summary of the Use Case Assessment

In our examples, the overall ability of ChatGPT to translate programming languages was successful and accessible without deeper knowledge of the target software. However, certain limitations are to be considered. While GPT‐4o was able to generate similar statistical models in different programming languages, it did not accurately translate the statistical details of the models. In both use cases (SPSS → R and R → Python), ChatGPT used simpler implementations, at times relying on existing implementations, instead of generating code that uses the same evaluation metrics. The majority of chats (80% in both Python and R translations) explained this discrepancy and offered to provide more truthful implementations. Furthermore, there are inherent differences between the programming languages, for example, regarding random number generators. That is why one should expect certain differences in the outcomes in non‐deterministic tasks or code that is heavily reliant on numerical operations. It should, however, be noted that some generated scripts were wrongfully stated to be accurate code translations, while in rare cases, the generated code documentation was incorrect. A certain degree of familiarity with the target language is thus recommended to verify the truthfulness of the translation. In most chats, the generated documentation was helpful for understanding the translation. All chats also offered short summaries of the generated code, as well as suggestions for further extensions, output metrics, and visualization tools.

A further caveat is the use of deprecated software packages or function argument names. These arose in both considered use cases. While all of these issues were resolved after providing ChatGPT with the resulting error messages, changes in parameter names or functionality of packages that do not result in errors might go unnoticed.

In summary, ChatGPT can be helpful in translating code into different programming languages, although slight adaptations and careful checking of implementation details might be necessary to ensure truthful translations.

*Human‐in‐the‐loop checkpoints*:

- *Package availability and deprecation*: ChatGPT generates solutions based on forum posts and software documentation that may be out of date. Users should verify that the suggested packages or modules are still available or might be deprecated, and check if functionality and syntax deviate from the usage suggested by ChatGPT.
- *Evaluation metrics*: ChatGPT does not generally consider differences in the default implementations of statistical methods across different programming languages, especially with regard to evaluation metrics and the tuning of hyperparameters. Users should therefore examine such possible differences and adjust the code as appropriate.
- *Generalizability*: With code involving randomly generated data or intended for use with multiple datasets, the generated translations may fail in certain input constellations. In these cases, users should run the generated code either multiple times or using different datasets to detect and debug potential issues.

## 4 Final Conclusion, Recommendations, and Discussion

This tutorial paper employs multiple use cases to demonstrate how generative AI can be integrated into the daily workflow of professional biostatisticians. While some tasks were completed rather satisfactorily, others suffered from severe issues. For instance, some meta‐analysis results appeared generally sound, yet not perfect. Their high variability across runs shows the inherent randomness of large language models. Note that variability can also occur when analyses are conducted by experienced human statisticians due to the multiplicity of possible meaningful analysis strategies [55]—see, for example, a recent multiverse meta‐analysis yielding very diverse, even conflicting, results [56]—since there is rarely a single correct solution for related statistical tasks. However, solutions proposed by ChatGPT in some of the runs were completely incorrect. In another use case, ChatGPT provided the correct methodological background and R scripts for the latent class analyses. To handle numerical problems when using these R scripts, the analysts had to perform specific changes in the central function and choose sensible parameters. Next, ChatGPT provided a helpful teaching session to understand individual‐level surrogacy and related numerical measures, explaining the relationship between *mutual information* and *likelihood reduction factor* with helpful examples and R scripts. However, sample size calculations were often underestimated across runs. Also, the statistical plots provided by ChatGPT exhibited deficiencies at times. In the causal inference use case, ChatGPT provided conceptually plausible explanations but struggled with key technical aspects, including misrepresenting causal relationships in a DAG and inadequately (or incorrectly) implementing inverse probability of censoring weights. Similar observations were made in the simulation use case, where ChatGPT struggled to choose exact confidence intervals. Moreover, it required multiple attempts to find a prompt resulting in somewhat sensible confidence intervals. Conversely, translation between programming languages typically worked well, although users must ensure that specific statistical model building is neither compromised nor reliant on deprecated software packages.

One important conclusion from these findings on ChatGPT is that numerous pitfalls exist within the details of statistical analyses—often inadequately addressed by LMMs. When it comes to such details, carefully supervising the AI's results and adjusting the prompts if needed, is of the essence. In particular, we strongly warn about the non‐skeptical use of LLMs. Relying naively on the correctness of their output is irresponsible, and statistical studies can be seriously corrupted. Consequently, expert knowledge from biostatisticians remains indispensable, along with maintaining a questioning stance towards AI outputs.

Despite these challenges, we still see great potential in the use of large language models by biostatisticians because they hold promise for saving a considerable amount of time. Although AI requires steering and oversight, it delivers rapid results while often self‐correcting upon request. *Prompt engineering*—structuring input prompts for optimal output—is particularly relevant for API users who enjoy greater flexibility; however, common chat interfaces also benefit from testing input order relevance: While there was hardly any influence on the results of sample size planning, the results of the simulation study were severely influenced by the wording and structuring of the prompt. One may speculate that ChatGPT had more learning material for the sample size use case compared to the less frequent simulation of coverage probabilities. This would at least match the failure to suggest exact confidence intervals.

Derived from the experience collected in the various use cases of this paper, we propose the following guidelines for biostatisticians when working with generative LLMs, in this case coined to OpenAI's GPT‐4o:

- utilize generative LLMs for expediting routine tasks or components thereof to save time;
- provide sufficient context to the LLM so as to thoroughly prepare it for the task;
- critically double‐check outcomes as the LLM does not always accurately reproduce or apply human expertise;
- even double‐check whether the results in the LLM's response text align with the results visible in the *data analysis mode*;
- rectify the LLM's results by confronting it with earlier flaws or making suggestions for improvements;
- be aware of the inherent randomness of the LLM; potentially re‐run analyses in multiple, independent chats to assess the variability and stability of its outputs;
- keep in mind the statistical results' dependence on the programming language used by the LLM;
- understand that the LLM evolves over time, which might call for adjusted strategies of usage.

We advocate training students to use LLMs effectively, e.g., in the context of programming, applied data analysis, or lectures introducing statistical methods and approaches. It is essential for users to recognize the limitations of LLMs and the necessity of critically validating their results, as outlined in the guidelines above. Ultimately, the responsibility for ensuring the correctness of a statistical analysis lies with the biostatistician. While LLMs can assist with certain tasks in biostatistics, they cannot replace the expert knowledge of a well‐trained biostatistician—this expertise is a crucial prerequisite for appropriate usage of LLMs.

In view of the newer ChatGPT models that have been released during the preparation of this tutorial, and also given the rapid advancement of large language models in recent years, we expect great improvements yet to come. It is possible that our assessment in the present paper will soon require revision. In particular, we observed that many (but not all) results seemed more promising when obtained from the newer models o3 or o4‐mini compared to 4o. However, the choice of whether to use *deep research* and/or *reasoning* can make a crucial difference. Additionally, just when the revision of this document was finalized, GPT‐5 was released on August 7, 2025. Based on these findings, we recommend users continuously reassess AI reliability as models evolve and improve. As with many papers in fast‐evolving fields, the present paper will become outdated quickly; however, we expect our core insights to remain relevant.

Note that we have not evaluated GPT‐4o's capabilities in writing, reviewing, and summarization, or their mathematical‐statistical abilities to prove assertions without logical flaws. These tasks are undoubtedly important skills for biostatisticians, but such investigations are beyond the scope of the present paper. Here, we mainly focused on various data analytical and statistical software‐related tasks. However, to briefly address ChatGPT's summarization and reviewing skills, we also investigated Editor Paul Albert's idea to let ChatGPT reflect on the present manuscript. That analysis, alongside screenshots of the corresponding chats, is part of the Supporting Information.

While our paper focuses on the potential applications of LLMs and their output accuracy, we have deliberately not explored other important aspects of their impact on the profession of statisticians—for instance, the broader implications for job satisfaction. The reduction of routine tasks through LLMs is likely to be seen as a positive development. However, recent research by Woodruff et al. [57]—though not specifically focused on biostatistics—suggests that increased AI usage among knowledge workers may also lead to negative consequences like deskilling, dehumanization, and disconnection. They also quote comments from software developers in their research workshop that might also apply to biostatisticians: “[…] some of us are motivated by our love of tinkering and problem solving, and generative AI may take over a lot of the work that brings us joy” or “There are folks who get their joy and their sense of meaning from writing code and that's kind of their thing […].” It is easy to imagine that, for some of us, reviewing analysis pipelines and software code does not provide the same sense of intellectual satisfaction as actively creating them. Nevertheless, similar to previous industrial revolutions, these advancements also necessitate adaptation and new skills. The challenge for future biostatisticians will be to leverage the considerable potential of LLMs while ensuring high‐quality work and maintaining job satisfaction.

## Disclosure

The authors have nothing to report.

## Conflicts of Interest

The authors declare no conflicts of interest.

## Policy on Using ChatGPT and Similar AI Tools

During the preparation of this manuscript, we also used the GPT‐4o model for minor language edits, aiming to enhance readability. After using this tool, the authors reviewed and edited the content as needed and take full responsibility for the content of the proposal.

## Supporting information




**Data S1.** Supporting Information.

## Data Availability

The data that support the findings of this study are openly available in ChatGPT‐as‐a‐Tool‐for‐Biostatisticians at https://github.com/dennis‐dobler/ChatGPT‐as‐a‐Tool‐for‐Biostatisticians.

## Endnotes

^1^

https://www.economist.com/technology‐quarterly/2024/03/27/can‐artificial‐intelligence‐make‐health‐care‐more‐efficient, https://www.economist.com/technology‐quarterly/2024/03/27/ais‐will‐make‐health‐care‐safer‐and‐better.

^2^

https://github.com/dennis‐dobler/ChatGPT‐as‐a‐Tool‐for‐Biostatisticians.

^3^

https://platform.openai.com/tokenizer, accessed March 10, 2025.

^4^

https://openai.com/index/improvements‐to‐data‐analysis‐in‐chatgpt/, accessed June 8, 2025.
