# Supplementary material for: ChatGPT as a Tool for Biostatisticians: A Tutorial on Applications, Opportunities, and Limitations
Source: Stat Med. 2025 Oct 23;44(23-24):e70263. doi: 10.1002/sim.70263 (PMC12548020; doi:10.1002/sim.70263)
Supplement: Supplementary file 1 — Data S1. Supporting Information. [file SIM-44-0-s001.zip › Supplementary Material Document tracked changes.pdf]

Received: Added at production | Revised: Added at production | Accepted: Added at production

DOI: xxx/xxxx

ARTICLE TYPE

Supplementary Material for "ChatGPT as a Tool for Biostatisticians: A Tutorial on Applications, Opportunities, and Limitations"

Dennis Dobler[1] | Harald Binder[4] | Anne-Laure Boulesteix[5,6] | Jan-Bernd Igelmann[2] | David Köhler[8] | Ulrich Mansmann[5] | Markus Pauly[2,3] | André Scherag[7] | Matthias Schmid[8] | Amani Al Tawil[5] | Susanne Weber[4]

[1]Department of Mathematics, RWTH Aachen University, North Rhine-Westphalia, Germany

[2]Department of Statistics, TU Dortmund University, North Rhine-Westphalia, Germany

[3]Research Center Trustworthy Data Science and Security, University Alliance Ruhr, North Rhine-Westphalia, Germany

[4]Institute of Medical Biometry and Statistics, University Medical Center Freiburg, Baden Württemberg, Germany

[5]The Institute for Medical Information Processing, Biometry, and Epidemiology, LMU Munich, Bavaria, Germany

[6]Munich Center for Machine Learning, Bavaria, Germany

[7]Institute of Medical Statistics, Computer and Data Sciences, Jena University Hospital - Friedrich Schiller University, Thuringia, Germany

[8]Institute for Medical Biometry, Informatics and Epidemiology, University Hospital Bonn, North Rhine-Westphalia, Germany

Correspondence
Dennis Dobler
Email: dennis.dobler@rwth-aachen.de
Note: apart from the corresponding author, all other authors were listed in alphabetical order.

Present address
Kreuzherrenstr. 2, 52056 Aachen, Germany.

Abstract

This supplementary material file contains the most relevant additional information related to the main manuscript. Additional supplementary materials are to be found in separate sub-folders. The present document foremost gives an overview of all these files but it also offers other insights: for example, additional figures, more extensive prompts (beyond the initial ones), and ChatGPT's evaluation of our main manuscript.

KEYWORDS

causal analysis, diagnostic accuracy, generative AI, individual-level surrogacy, large language model, latent class analysis, meta-analysis, sample sizes planning, simulation study, translation programming languages

5 | GENERAL NOTES

Since each use case has been independently repeated ten times, there are ten files per use case repesenting the chat with ChatGPT-4o. Although it is not apparent from the chats exported to PDF files, all use cases employed the *data analysis mode* of ChatGPT. The data sets and software code generated from ChatGPT's *data analysis mode* were uploaded to a Github repository

Abbreviations: LLM, large language model.

 © 2023 Copyright Holder Name

for reproducibility reasons.[‡] Note that these files are not part of the Supplementary Materials available online on the Statistics in Medicine webpage.

5.1 | Comments on the Variation in the Independently Repeated Chat Sessions

In each use case, the team produced ten ChatGPT reactions on the same set of prompts. There was variability within each set of answers which may be of interest from a statistical or analytical perspective. Several types of insights could be derived.

First, one can learn about epistemic variability (answer variance). Variations may suggest that the questions are ambiguous, ChatGPT's knowledge is broad but not definitive on the topic, or the subject matter allows for open interpretation (methodological creative questions). This allows to gauge the epistemic uncertainty or answer stability of the language model. A second point relates to semantic clustering. This is related to the point of how many distinct perspectives ChatGPT offers on a single question. One could explore whether the ten answers form distinct clusters (e.g., conservative vs. speculative, technical vs. conceptual). Third, the variability elucidates stochastic behavior. Since ChatGPT operates probabilistically (unless temperature = 0), one could analyze: How much randomness influences the response, how different temperature settings affect answer diversity. This would allow to explore the relationship between model temperature, prompting, and output variability. The fourth issue is robustness and bias detection. By comparing the responses, one can identify systematic tendencies or biases in how the model frames its answers. It is of interest whether certain perspectives are consistently favored. The fifth point relates to prompt engineering sensitivity. The material can be used to test how sensitive the model is to small changes in the prompt, which is crucial for prompt engineering. It would be of interest to determine where the boundary lies between stable and volatile model outputs.

We did not systematically explore the five mentioned issues; they are left for future research. The results may be of interest to shape teaching approaches on how to use ChatGPT, to learn how certain or uncertain the model is on a topic, what conceptual or interpretive directions it offers, how randomness plays into response generation, and whether it shows consistent biases or stable tendencies. However, the additional file *5.1_Variability_in_Chats.pdf* is GPT-4o's response to the first paragraph in this subsection when asked for a biostatistical example for the points mentioned above; it provides sensible suggestions and a summarizing table at the end.

6 | SUPPLEMENT FOR USE CASE: SYSTEMATIC REVIEWS AND META ANALYSES

In addition to the analyses and results presented in the main paper, this supplement contains multiple additional files in the folder *Use_Case_Systematic_Reviews_Meta_Analyses*: screenshots of four times ten independent ChatGPT chats for data extraction or systematic review part (GPT-4o and prompt with reasoning, GPT-o4-mini and prompt with reasoning, GPT-o4-mini and base prompt, GPT-o4-mini and base prompt) and two times of ten independent chats for conducting the meta analyses (GPT-o4 mini and prompt with reasoning, GPT-4o and base prompt) alongside the corresponding forest plots from these two settings.

6.1 | Prompts of the Systematic Review Section

The following is the initial base prompt that has been used for the data extraction.

> **Prompt:**
> *The aim is to perform a meta-analysis on total ipsilateral local recurrence rates at 10 years in cases of ductal carcinoma in situ*
> *Data inclusion rules are:*
> *To ensure the validity of the follow-up data, the results should be as close to 10 years follow-up (minimum) as the information in each study allows. If there is more than one publication from an institution or group, the most recent study with appropriate follow-up will be used to extract 10-year data.*

[‡] https://github.com/dennis-dobler/ChatGPT-as-a-Tool-for-Biostatisticians

Please provide:

- *the rate of ipsilateral local recurrence for conservative surgery with additional radiotherapy (no other interventions or drugs allowed) after 10 years*
- *the number of eligible patients*
- *period of data collection*
- *duration of follow-up*
- *type of study*
- *age of patients (mean or median, maximum and minimum)*
- *treatment modalities*

(Please provide a data frame with one entry for each study, and column names "study", "rate", 'n_patient', 'period', 'follow_up', 'study_type', 'age', 'treatment'. Do not provide additional text).

The second prompt that was used is the following one.

Prompt:
The aim is to perform a meta-analysis on total ipsilateral local recurrence rates at 10 years in cases of ductal carcinoma in situ.
Data inclusion rules are: To ensure the validity of the follow-up data, the results should be as close to 10 years follow-up (minimum) as the information in each study allows. If there is more than one publication from an institution or group, the most recent study with appropriate follow-up will be used to extract 10-year data. Please provide from each of the two attached studies:

- *the rate of ipsilateral local recurrence for conservative surgery with additional radiotherapy (no other interventions or drugs allowed) after 10 years*
- *the number of eligible patients*
- *period of data collection*
- *duration of follow-up*
- *type of study*
- *age of patients (mean or median, maximum and minimum)*
- *treatment modalities*

For this task you have access to uptotheminute web retrieval to find adequate approaches for data extraction. When answering the task above please proceed as follows:

1. *Fetch the most relevant sources.*
2. *Show your stepbystep reasoning.*
3. *At the end, give me a concise answer citing the sources you used.*
4. *Please provide a data frame with one entry for each study, and column names 'study', 'rate', 'n_patients', 'period', 'follow_up", 'study_type', 'age', and 'treatment'.*

6.2 | Results of Chat GPT-4o's Meta Analysis

In the following, the results of the initial Chats that were performed based on a shorter prompt and carried out with Chat-GPT-4o are presented. The prompt used was as follows.

Prompt: *'The goal is to perform a subset meta-analysis for the given data set. The target variable is the local recurrence rate. Create a subset for all treatment types. Decide all methodological questions yourself (fixed effect vs. random effects, etc.). Follow clearly formulated rules for selecting the model type. Please provide a (subset) forest plot and all associated confidence*

intervals. Do not forget the pooled overall estimate and its confidence interval.'

Each of the ten runs produced distinct meta-analysis results and no run fully aligned with our preferred analysis approach. The pooled estimates for each treatment and applied transformations alongside model selections for all chats are detailed in Table 1.

Key findings of the ten runs are as follows: in 9 out of 10 runs, GPT-4o correctly identified the hierarchical structure of the dataset; a logit transformation was applied three times. The transformation simplifies the handling of 0 event studies and prevents negative estimates or CIs which appeared in some of the chats that did not apply a transformation.

GPT-4o provided both FE and RE results together with a recommendation for model selection (FE vs. RE) typically based on the heterogeneity statistic I^2—the percentage of variability attributed to heterogeneity. In eight cases it suggested an RE model due to $I^2 > 0.5$; one chat used $I^2 > 0.4$ as a cutoff while a Q-test informed one decision.

Nevertheless, some runs produced fairly accurate results. An example of a rather correct first-try analysis is the forest plot from Chat 2 in Figure 1 (left). Here, the LLM performed a logit transformation along with nearly-correct subgroup analyses that would have led to the same conclusions. In contrast, Figure 1 (right, from Chat 5) illustrates a very problematic result, where no rate transformation was applied and the computed CIs included implausible negative values. Furthermore, in Chat 4, even negative point estimates were given after ChatGPT stated that only an unspecified continuity correction for zero events had been performed. Thus, the user may be fortunate and receive a fairly accurate result (as in Run 2), or encounter a incorrect one (as in Run 4 or 5).

Another key observation was inconsistency in accurately reporting effect estimates from the forest plots presented in the chats. While some chats provided correct values directly, others required multiple prompts with varying specificity; in some cases, no valid estimates were obtained at all. For example, in Chat 3, correct estimates were retrievable only after several follow-up requests, whereas in Chat 6, no meaningful values were provided. In Chat 8, although all five rates were displayed in the forest plot (and used for Table 8), the chat output included only the three values $(0.0142, 0.0929, -4.2546)$, labeled as "five-dimensional vector" and containing implausible values, such as a large negative rate. Notably, the overall pooled estimate (across all therapies) was omitted in Chats 3, 5, and 6. Furthermore, the assignment of estimates to the four specific therapies was not always coherent. For instance, in Chat 5, the estimates for Mastectomy and Biopsy-Only were reversed, and likely also in Chat 10, where Table 1 reports the subset estimates explicitly stated in the chat but the forest plot hints at a flip.

TABLE 1 Obtained effect estimates for each treatment and each of the ten GPT-4o chats together with information about the usage of a transformation and the decision between RE and FE. In addition, the last row shows a re-analysis using the logit transformation and an hierarchical RE model structure.

Pooled est.	Mastectomy	Surg. & radiation	Surg. no radiation	biopsy-only	overall	Rate transf.	RE/FE	decision criterium
Chat 1	0.02	0.06	0.11	0.25	0.08	-	mixed	$I^2 \geq 0.5$
Chat 2	0.02	0.07	0.11	0.25	0.08	logit	mixed	Q-test
Chat 3	0.00	0.07	0.11	0.25	NA	-	mixed	$I^2 > 0.5$
Chat 4	0.00	0.04	0.09	-0.47	-0.08	continuity correction	mixed	$I^2 > 0.5$
Chat 5	0.00	0.06	0.11	0.25	NA	-	mixed	$I^2 \geq 0.5$
Chat 6	NA	NA	NA	NA	NA	logit	mixed	$I^2 \geq 0.5$
Chat 7	0.01	0.04	0.06	0.08	0.08	-	mixed	$I^2 \geq 0.5$
Chat 8	0.01	0.04	0.06	0.09	0.01	logit	mixed	$I^2 > 0.5$
Chat 9	0.01	0.07	0.11	0.24	0.11	-	mixed	$I^2 \geq 0.4$
Chat 10	0.25	0.06	0.11	0.01	0.07	-	mixed	$I^2 \geq 0.5$
Our analysis	0.03	0.07	0.11	0.25	0.08	logit	RE	-

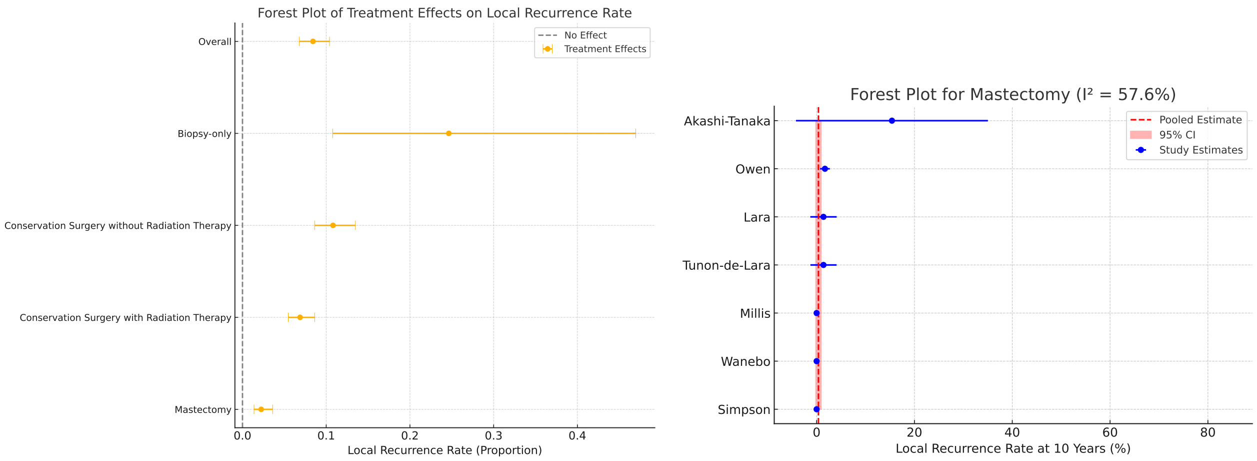

FIGURE 1 Forest plots of Chat 2 (well analyzed, treatment level) and Chat 5 (erroneously analyzed, studies for Mastectomy) in the reanalysis of the meta-analysis of Stuart et al.[1] via GPT-4o.

7 | SUPPLEMENT FOR USE CASE: LATENT CLASS ANALYSIS – UNKNOWN GOLD STANDARD IN DIAGNOSTIC QUALITY ASSESSMENT

The supplement to this use case is located in the folder *3.2_Diagnostic_Test*; it contains the screenshots of the ten chats. The Github repository contains all ten R scripts generated by ChatGPT.

8 | SUPPLEMENT FOR USE CASE: INDIVIDUAL-LEVEL SURROGACY IN THE CONTEXT OF INFORMATION THEORY

The supplement to this use case is located in the folder *3.3_Individual-Level_Surrogacy*; it contains the screenshots of the ten chats. The Github repository contains all ten R scripts generated by ChatGPT.

9 | SUPPLEMENT FOR USE CASE: SAMPLE SIZES PLANNING FOR SURVIVAL OUTCOMES

The supplement to this use case is located in the folder *3.4_Sample_Sizes_Planning_for_Survival_Outcomes*. Next to the screenshots of the ten chats stored in the folder *3.4_Chats*, the folder *3.4_Sample_Sizes_Newer_Versions* contains screenshots of the additional chats described in the following subsection. The Github repository contains all ten R scripts and ten data sets generated by ChatGPT.

The detailed prompt guidelines (also beyond the initial prompt) are as follows:

Prompt:

- *'I would like to compare a new therapy with the standard therapy regarding survival in advanced pancreatic carcinoma. Please perform a sample size calculation and incorporate the following parameters:*

  - *Primary outcome: Overall survival.*
  - *Median survival time in the standard therapy group: 12 months.*
  - *Median survival time in the new therapy group: 18 months.*
  - *Power* = 80% ($1 - \beta = 0.8$)
  - *a two-sided significance level of* $\alpha = 0.05$.
  - *The allocation ratio should be 1:1 (equal group sizes).*

- · *Patients will be followed up for 2 years.*
- · *We expect a dropout rate of 15%.*

Use the data analysis mode.
- *(if not already explained in detail) please explain how you calculated the sample size.*
- *(If needed) please check whether the formular for the required number of events is correct. Did you incorporate the proportions of group size?*
- *Please draft a suitable section on sample size calculation for the study protocol.*
- *Please simulate data accordingly. Incorporate the dropout rate and that patients are censored at the end of follow-up*
- *Please visualize the data*
- *(after visual evalutation of curves: correction if needed, eg.) Kaplan Meier curves should start at (0,1)*
- *Please compare the groups using the log-rank test.*
- *Please take meaningful relationships with other covariates into account and simulate corresponding data. Remember the disease setting of pancreatic carcinoma*
- *(if not incorportated: add ECOG groups to the data)*
- *Please explain the associations you considered*
- *Please show me kaplan meier curves for the treatment comparison in ECOG groups*
- *(if needed) please show one plot per ECOG group*
- *Please perform a Cox regression for the treatment comparison. Do it first without further adjustment. In a next step do it with adjustment for further covariates. Present the results in a table with one row per covariate and the following columns: variable| HR unadj. | 95% - CI | p-value| | HR adj. | adj. 95%-CI|adj. P-value*
- *Usually I am using R. please provide me the corresponding code to the presented plots and analysis.*
- *Provide me the final dataset as download'*

10 | SUPPLEMENT FOR USE CASE: CAUSAL INFERENCE AND THE USE OF IPCW TO ADJUST FOR TREATMENT SWITCHING

The supplement to this use case is located in the folder *3.5_Causal_Inference*; it contains the screenshots of the ten chats. The detailed prompt guidelines (also beyond the initial prompt) are as follows:

Prompt:
- *'I have data from a randomised clinical trial. I want to compare the efficacy of the experimental treatment with that of the control on overall survival in patients with non-small cell lung cancer. In this two-arm RCT, a considerable number of patients in the control arm switched to the experimental treatment after disease progression, while those in the experimental arm never switched.*
  My goal is to estimate the causal effect of sustained intake of the experimental treatment versus sustained intake of control on Overall survival, had the control group patients not been allowed to switch. Based on the information provided above, please define my target Estimand, following the guidance from the Estimand framework (ICH-E9 addendum).
- *The switching behaviour is affected by a set of different baseline and time-varying covariates. Here, I want to assume that the time-varying confounders that influence the switching behaviour and Overall survival are also themselves affected by prior treatment intake. For e.g. ECOG score might not only affect whether patients should switch treatment or not, but its value might also be affected by prior treatment intake reflected by the switching behaviour. In this case, the analytical problem becomes more complex. Can you explain?*
- *Depict our research problem in a suitable DAG*
- *I would like to apply the inverse probability of censoring weighting (IPCW) approach to address the bias introduced by switching following the methodology discussed in the following paper: Robins JM, Finkelstein DM. Correcting for noncompliance and dependent censoring in an AIDS clinical trial with inverse probability of censoring weighted (IPCW) log-rank tests. Biometrics. 2000;56(3):77988.*
  Briefly summarise the key points from this paper.
- *Could you elaborate more on the research question Robins and Finkelstein were trying to answer in their paper, mentioning the comparator arms, possible intercurrent events, outcome definition and population under study.*

- *Attached, I provide you with a directed acyclic graph (DAG) that represents our research problem. To simplify this DAG and take into consideration this deterministic information provided below, I restrict my DAG to the post-progression period for patients in the control arm only and consider two follow-up time points, 1 2. Here A(k) is deterministically 0 if A(k-1)=0 AND L(k-1) (reflecting progression status)=0 (no progression). Following disease progression, the decision as to whether or not a patient in the control group continues to take their treatment (A0 or A1) depends on predictive factors measured at or before the time of disease progression (L0). Again, we assume that treatment intake (A0) affects outcome (Y) and influences a set of TVCS at a later time-point (L1). These covariates are also assumed to impact the clinicians decision as to whether or not a patient should subsequently change treatments (A1), introducing time-varying confounding and treatment confounder feedback through L1. I will emphasise again that in our case scenario, patients in the control group do not switch unless they progress, and all patients in the experimental group never switch. I still want to estimate in my outcome model the causal effects of sustained treatment interventions (always taking control versus always taking experimental) under a hypothetical strategy where control patients do not switch treatment. I want you to use this DAG as a basis for performing the appropriate IPCW analyses and provide me with the analysis R-script. You may assume that the dataset is in long format, with variables: id, time (in months and increases with increments of 1), death indicator, switching indicator, baseline covariates repeated over time points, time-varying covariates at each time pt.'*

11 | SUPPLEMENT FOR USE CASE: SIMULATION STUDY

The supplement to this use case is located in the folder *3.6_Simulation_Study*; it contains the screenshots of the ten chats corresponding to GPT-4o and of the three chats corresponding to GPT-o3 + *deep research*.

12 | SUPPLEMENT FOR USE CASE: TRANSLATION BETWEEN MULTIPLE STATISTICAL PROGRAMMING LANGUAGES

As this use case consists of two separate considerations (translation from R to Python and also from SPSS to R), the folder *Use_Case_Translation_Between_Multiple_Statistical_Programming_Languages* also contains two corresponding subfolders. Each of these two contain the subfolders *Chats* (with all ten ChatGPT conversations) and the original code (in R and SPSS, respectively). The chat-specific translations to Python and R, respectively, are available on the Github repository. The Github repository contains all ten translated Python scripts (and additionally six updated files) and ten translated R scripts (and additionally eight updated files) generated by ChatGPT.

13 | CHATGPT'S REVIEW OF OUR MANUSCRIPT

Inspired by similar examples provided by Editor Paul Albert, we have also asked ChatGPT to evaluate the original submission of the main paper from its own perspective. We have used the following prompt for different versions of ChatGPT; here, we only present the results from GPT-4o and GPT-o3 because 4o was used throughout the paper and o3 seemed to give the most interesting output.

> **Prompt:** *'What does ChatGPT think about the attached manuscript entitled "ChatGPT as a Tool for Biostatisticians: A Tutorial on Applications, Opportunities, and Limitations"? Provide a high-level evaluation as if written by ChatGPT itself - highlighting what I (as ChatGPT) would think about the content, structure, and portrayal of LLMs in the manuscript.'*

For each model, we ran each prompt three times in independent chat instances. Screenshots of the six chats are included in the additional files of the Supplementary Materials.

We found all evaluations originating from GPT-4o to be generally positive and not very skeptical; they gave a benevolent summary of our paper, using different categories in their presentations. They only mentioned one to four points for improvement. Our portrayal of ChatGPT or LLMs was consistently summarized as balanced, fair, insightful, detailed, and/or realistic. ChatGPT stressed our criticism and praise of its performance in the different use cases.

The evaluation of GPT-o3 seemed more varied and diverting: the potential points for strengthening our paper seemed more sophisticated and to the point and it took more of a skeptical stance although its general impression is still quite positive; it even showed signs of irony. All of this suggests that the model GPT-o3 may be better suited for such evaluations. However, since such research questions were not central to our paper, we did not explore this further.

We would also like to point to the preprint by Needham *et al.*[2] They investigated the *evaluation awareness* of LLMs by asking the models for each transcript in the dataset: "Was this from an evaluation?". For example, based on chat settings, GPT-4.1 scored an AUC of 0.77 for the detection of an evaluation setting. It is conceivable that LLMs that identified an evaluation setting respond differently.

REFERENCES

1. Stuart KE, Houssami N, Taylor R, Hayen A, Boyages J. Long-Term Outcomes of Ductal Carcinoma in Situ of the Breast: A Systematic Review, Meta-Analysis and Meta-Regression Analysis. *BMC Cancer.* 2015;15(1):890. doi: 10.1186/s12885-015-1904-7
2. Needham J, Edkins G, Pimpale G, Bartsch H, Hobbhahn M. Large Language Models Often Know When They Are Being Evaluated. *arXiv preprint arXiv:2505.23836.* 2025.
